# Immune Training of the Interleukin 6 Gene in Airway Epithelial Cells is Central to Asthma Exacerbations

**DOI:** 10.1111/all.70070

**Published:** 2025-10-16

**Authors:** Lars P. Lunding, Markus Weckmann, Ulrich M. Zissler, Constanze Jakwerth, Rebecca Bodenstein‐Sgró, Sina Webering, Christina Vock, Johanna C. Ehlers, Romina A. M. Fernandez Ceballos, Sai Sneha Priya Nemani, Karosham Diren Reddy, Brian George G. Oliver, Cornelis J. Vermeulen, Maarten van de Berge, Carole Ober, Axel Künstner, Hauke Busch, Inke König, Christoph Garbers, Carsten B. Schmidt‐Weber, Marcel F. Nold, Ali Önder Yildirim, Claudia A. Nold‐Petry, Zane Orinska, Thomas Bahmer, Jan Heyckendorf, Gesine Hansen, Erika von Mutius, Klaus F. Rabe, Anna‐Maria Dittrich, Bianca Schaub, Folke Brinkmann, Matthias V. Kopp, Michael Wegmann, The ALLIANCE Study Group as part of the German Centre for Lung Research (DZL)

**Affiliations:** ^1^ Division of Lung Immunology Priority Area Chronic Lung Diseases, Research Centre Borstel ‐ Leibniz Lung Centre Borstel Germany; ^2^ Airway Research Centre North Member of the German Centre for Lung Research (DZL) Germany; ^3^ Department of Paediatric Pneumology & Allergology University Clinics Schleswig‐Holstein (UKSH) Lübeck Germany; ^4^ Division of Epigenetics of Chronic Lung Diseases Priority Area Chronic Lung Diseases, Research Centre Borstel ‐ Leibniz Lung Centre Borstel Germany; ^5^ Centre of Allergy and Environment (ZAUM) Technische Universität and Helmholtz Centre Munich Munich Germany; ^6^ Technology Transfer Center for Building Biology and Indoor Health Rosenheim Technical University of Applied Sciences Freilassing Germany; ^7^ Department of Medicine, Pulmonary and Critical Care Medicine, University Medical Center Giessen and Marburg, Philipps‐University Marburg, German Center for Lung Research (DZL) Universities of Giessen and Marburg Lung Center (UGMLC) Marburg Germany; ^8^ Comprehensive Pneumology Centre Munich (CPC‐M) Member of the German Centre for Lung Research (DZL) Germany; ^9^ Division of Experimental Pneumology Priority Area Chronic Lung Diseases, Research Centre Borstel‐ Leibniz Lung Centre Borstel Germany; ^10^ School of Medical and Molecular Biosciences University of Technology Sydney Australia; ^11^ Woolcock Institute of Medical Research The University of Sydney Australia; ^12^ University Medical Centre Groningen Department of Pulmonary Diseases, GRIAC the Netherlands; ^13^ Department of Human Genetics University of Chicago Chicago Illinois USA; ^14^ Luebeck Institute of Experimental Dermatology and Institute for Cardiogenetics University of Luebeck Luebeck Germany; ^15^ Luebeck Institute of Experimental Dermatology, Institute for Cardiogenetics and Centre for Research of Inflammatory Skin Disease (CRIS) University of Luebeck Luebeck Germany; ^16^ Institute for Medical Biometry and Statistics University of Luebeck Luebeck Germany; ^17^ Instititute for Clinical Biochemistry Hannover Medical School Hannover Germany; ^18^ Department of Paediatrics, Monash University Melbourne Victoria Australia; ^19^ Ritchie Centre, Hudson Institute of Medical Research Melbourne Victoria Australia; ^20^ Monash Newborn, Monash Children's Hospital, Melbourne Victoria Australia; ^21^ Institute of Lung Health and Immunity (LHI) Helmholtz Munich Munich Germany; ^22^ Institute of Experimental Pneumology, LMU University Hospital German Ludwig‐Maximilian's University Munich Germany; ^23^ Division of Immunology and Cell Biology Priority Area Chronic Lung Diseases, Research Centre Borstel ‐ Leibniz Lung Centre Borstel Germany; ^24^ University Hospital Schleswig‐Holstein Campus Kiel Department for Internal Medicine I Kiel Germany; ^25^ Translational Paediatric Pneumology, Department of Paediatric Pneumology Allergology and Neonatology, Hannover Medical School Hannover Germany; ^26^ Biomedical Research in End Stage and Obstructive Lung Disease Hannover (BREATH) Member of the German Centre of Lung Research (DZL) Germany; ^27^ Institute for Asthma and Allergy Prevention, Helmholtz Centre Munich German Research Centre for Environmental Health Neuherberg Germany; ^28^ Dr. von Hauner University Children's Hospital of Ludwig Maximilian's University Munich Germany; ^29^ Department of Pneumology LungenClinic Grosshansdorf Grosshansdorf Germany; ^30^ Department of Paediatric Respiratory Medicine, Inselspital, University Children's Hospital of Bern University of Bern Bern Switzerland

**Keywords:** asthma, exacerbations, IL‐6, immune training, respiratory viruses

## Abstract

**Question:**

Epidemiological studies suggest that respiratory viral infections are major triggers of asthma exacerbations, and clinical studies have suggested the involvement of an increased interleukin‐6 (IL‐6) release. What is the pathophysiological role of IL‐6 in asthma exacerbation, and which mechanisms lead to enhanced IL‐6 release?

**Materials and Methods:**

Exacerbations of ovalbumin‐induced experimental allergic asthma were elicited in wild‐type and IL‐6‐deficient mice by intranasal (i.n.) application of poly(I:C). Airway inflammation, cytokine expression and release, mucus production and airway hyperresponsiveness were measured. IL‐6 was neutralised by i.n. anti‐IL‐6 antibody application. The human bronchial epithelial cell line, BEAS‐2B, was stimulated with poly(I:C) and infected with human rhinovirus‐16 in vitro, followed by quantification of *IL6* gene expression and DNA methylation. Genome‐wide DNA methylation was assessed in bronchial epithelial cells from adults with asthma (cohort I, *n* = 54) and in nasal epithelial cells from children and adults in the All‐Age‐Asthma cohort (ALLIANCE, *n* = 53 and *n* = 108 respectively).

**Results:**

Poly(I:C)‐induced experimental exacerbations in mice were preceded and paralleled by exaggerated IL‐6 release in the airway epithelium, with IL‐6 neutralisation completely preventing experimental exacerbations. Repetitive infection/stimulation with RV16 or poly(I:C) resulted in training of the IL‐6 release in human respiratory epithelial cells. In patients, hypomethylation at the *IL6* gene methylation was associated with high *IL6* expression and future exacerbations.

**Answer:**

An exaggerated IL‐6 release is required for exacerbation of experimental asthma, potentially the result of viral PAMP‐induced immune training of airway epithelial cells. Additionally, patients with asthma carrying the epigenetic signature of a trained IL‐6 response exacerbate more frequently. These findings open new avenues to identify and treat exacerbation‐prone patients.

## Graphical abstract

This study aims to investigate how immune activation influences the epigenetic regulation and expression of the Interleukin‐6 (IL‐6) gene during asthma exacerbations. By examining molecular mechanisms of immune training, we seek to elucidate how IL‐6‐driven inflammatory pathways contribute to airway dysfunction and worsening asthma symptoms. We deployed a multilevel, translational pipeline to identify the role of IL‐6, its gene methylation and downstream signalling in a murine model of experimental asthma exacerbation, in vitro tissue culture models and human cohorts. Murine models triangulated the central role of IL‐6 expression by using IL6 knockouts and mimicked a therapeutic approach using a neutralising antibody. The tissue culture models were conducted using a bronchial epithelial cell line (BEAS‐2B), and primary nasal and bronchial epithelial cells. In BEAS‐2B cells, repeated viral infections or stimulations with poly(I:C) resulted in increased IL‐6 release and epigenetic training was observed. Primary epithelial cells were used to confirm the effects of DNA methylation (DNAm) on IL‐6 transcription and secretion. Human cohorts were used to establish the molecular intercorrelation of DNAm, IL‐6 expression and IL‐6 trans‐signalling (IL6TS) in asthmatics. Nasal and bronchial specimens served to prove the transferability of the intercorrelation of DNAm and IL6TS between upper and lower airways. Nasal specimen data were used to identify clinical outcomes (exacerbations) to confirm the mouse model findings. In the paediatric cohort, a 36‐month follow‐up design allows for first associations with disease trajectory outcomes. This study investigates how immune activation influences the epigenetic regulation and expression of the IL‐6 gene during asthma exacerbations. Poly(I:C)‐induced excessive IL‐6 release, driven by training of innate immunity in the respiratory epithelium, is essential for the exacerbation of experimental asthma. Repeated RV16 or poly(I:C) exposure trained IL‐6 release in human respiratory epithelial cells. Asthma patients with a trained IL‐6 response, characterised by IL6 gene hypomethylation, exhibit more frequent exacerbations.

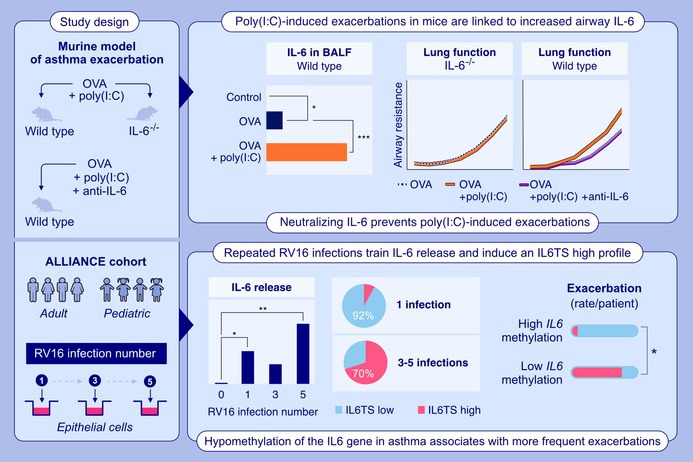

**Abbreviations**
ALLIANCE: All‐age asthma cohort
BALF: bronchoalveolar lavage fluid
IL: interleukin
IL6TS IL‐6: trans‐signalling
OVA: ovalbumin
Poly(I:C): polyinosinic: polycytidylic acid
RV16: rhinovirus‐16

## 1 Introduction

Asthma exacerbations occur in all asthma phenotypes, but particularly in patients with severe or difficult‐to‐treat asthma despite intensified therapies, resulting in increased utilisation of healthcare resources [1, 2]. The most common cause of exacerbations is infection by respiratory viruses, such as human rhinoviruses (RV), respiratory syncytial viruses and corona viruses [3]. Although these can trigger symptoms in many patients with asthma, it remains unclear why only some experience frequent exacerbations [4, 5]. Host defence mechanisms, including the epithelial release of the alarmin interleukin (IL) 25, chemokines, type I/III interferons and activation of the ER‐resident adaptor protein, stimulator of interferon genes (STING) or of the retinoic acid–inducible gene I (RIG‐I) inflammasome, have been reported to facilitate worsening asthma symptoms [6, 7, 8, 9]. Recently, two recent studies in patients with asthma suggested the involvement of interleukin‐6 (IL‐6) in the pathomechanism underlying exacerbations: In the first study, patients with high serum IL‐6 (> 1 pg/mL) had an increased exacerbation risk, whilst the second study demonstrated that a bronchial IL‐6 trans‐signalling (IL6TS) high expression profile, indicative of increased IL‐6 release, was also associated with more exacerbations [10, 11]. IL‐6 signalling occurs through various ways: Classical signalling, trans‐signalling and cluster signalling. In contrast to classical and cluster IL6 signalling, IL6TS occurs when IL‐6 binds to its soluble receptor (sIL‐6R), allowing signal transduction through membrane‐bound gp130‐expressing cells. This mechanism is implicated as a predominant pathway by which IL‐6 promotes (chronic) inflammatory diseases, making it a key target for therapeutic interventions [11, 12]. Although IL‐6 and other host defence mechanisms are not necessarily associated with reoccurring exacerbations, sentinel cells such as bronchial epithelial cells (BEC) remember previous contact with pathogenic compounds. This memory, in turn, alters their response upon reinfection, a mechanism referred to as ‘trained immunity’ [13, 14].

For the present study, we hypothesised that exaggerated IL‐6 release in response to repeated respiratory viral infection is central to the development of asthma exacerbations.

## 2 Materials and Methods

### 2.1 In Vivo Animal Studies

Female wild‐type C57BL/6, IL‐6‐deficient (IL‐6^−/−^) [15] and IL‐6 reporter (Il6^tm3307(Cerulean‐P2A‐CD90.1)Arte^) mice (*n* = 8 per group, unless stated otherwise), aged 6–8 weeks, were housed under specific pathogen‐free conditions. All animal studies were in accordance with the German animal protection law and were approved by the local animal research ethics board (V244‐230826/2015 [112–8/15]). Experimental allergic asthma (EAA) and subsequent exacerbations were induced as described previously [16] and in the supplement. For IL‐6 neutralisation, an anti‐IL‐6 monoclonal antibody was delivered via oropharyngeal application on Day 28. For repeated poly(I:C) exposures, weekly allergen aerosol challenge was continued for four additional weeks, receiving poly(I:C) on Days 35, 42, 49 and 57. For poly(I:C) dosage experiments, mice received 0, 2, 20 or 200 μg poly(I:C) on Day 28. Sampling for kinetic experiments was performed at 2, 4, 8 and 12 h after the last poly(I:C) application; all other experiments were sampled at 24 h. Negative controls were sham sensitised to phosphate‐buffered saline (PBS) and subsequently challenged with ovalbumin aerosol and were treated with PBS. For a detailed description, refer to the supplement.

### 2.2 In Vitro Studies in Human Bronchial Epithelial Cells

Human Bronchial epithelial cell line, BEAS‐2B (CRL‐9609) was commercially purchased from ATCC. The cells were then infected with human rhinovirus‐16 (RV16) as previously described [17] and in the supplement. Primary diseased human bronchial epithelial cells (DHBEs) from five donors with asthma (Table S1) were stimulated in vitro with poly(I:C) and recombinant human IL‐13. For details, see the Supporting Information.

### 2.3 Ex Vivo Studies

#### 2.3.1 Cohort I: Airway Epithelial Cells

Airway epithelial cells (AEC) samples were derived from 54 asthmatic subjects recruited in previous studies [18]. Endobronchial brushings were collected during bronchoscopy at the University of Chicago. Asthmatic subjects were included based on a current doctor's diagnosis of asthma, and they had no coexisting pulmonary conditions. All patients were using prescribed asthma medication at the time of sampling. Bronchial brushings were obtained as described previously [18, 19]. The clinical characteristics of Cohort I are presented in Table S2. DNA methylation, transcriptomic and clinical data from this previously described cohort [18, 19] were used for analysis in the present work. These studies were approved by the University of Chicago IRB protocol #09–421‐B and #15361A.

#### 2.3.2 ALLIANCE Cohort: Paediatric and Adult Arm

The All‐Age‐Asthma‐(ALLIANCE) paediatric cohort inclusion criteria comprised individuals aged 6–18 years, delivered at term (≥ 37 weeks), an active/passive understanding of German and had doctor‐diagnosed asthma (at age ≥ 6 years) according to current GINA guidelines. Nasal brushing samples were included from 90 paediatric participants that had sufficient DNA, with 3‐year follow‐up data available for 53 patients (Figure S1). The demographic characteristics of the paediatric cohort are presented in Table 1.

**TABLE 1:** Demographics of alliance paediatric cohort.

*IL6* gene methylation	Baseline		Follow‐up 1		Follow‐up 2		Follow‐up 3	
	Low (*n* = 34)	High (*n* = 19)	Low	High	Low	High	Low	High
Atopy (%)	70	89	72	82	76	82	76	92
ICS (%)	62	73	57	47	62	53	61	32^#^
Exacerbation (no.)	2	0	2	0	1	0	2	0
Tot. IgE (IU/mL)	283 (285)	416 (256)	262 (370)	720 (1555)	338 (515)	739 (1351)	400 (606)	344 (234)
Eosinophils (per dL)	433 (342)	512 (415)	366 (220)	431 (262)	414 (265)	366 (249)	595 (448)	227 (106)**
FEV1 (z‐score)	−0.05 (1.12)	−0.65 (0.75) ^§^	−0.25 (1.14)	−0.42 (0.56)	0.00 (1.12)	−0.36 (0.72)	−0.20 (1.26)	−0.32 (1.08)
FEV1/FVC (z‐score)	−0.42 (1.09)	−0.99 (1.16)	−0.33 (1.22)	−1.01 (1.95)*	−0.22 (1.27)	−0.86 (2.26)	−0.17 (1.40)	−1.09 (0.97)*
Controlled (%, GINA)	47	47	53	58	64	76	50	50

*Note:* At baseline: Females (%) 44 versus 21 (high vs. low *p* < 0.05), age (years) 9.52 versus. 11.82 (high vs. low, *p* < 0.05). Consecutive, yearly follow‐up.

Abbreviations: FEV_1_, forced expiratory volume in 1 s; FVC, forced vital capacity; ICS, inhaled corticosteroid.

* *p* < 0.05.

** *p* < 0.01 (low vs. high).

^#^ *p* < 0.05 (Follow‐up 3 vs. BL).

^§^ *p* = 0.057; Age, females (percentage of total number of children), total IgE, eosinophils (per dL), FEV_1_ (z‐score) and FEV_1_/FVC (z‐score) are displayed as mean. Values in brackets are standard deviations.

The ALLIANCE adult cohort includes individuals aged ≥ 18 years with an active/passive understanding of the German language. Adult healthy controls were individuals without any existing pulmonary disease. Nasal brushing samples were included from 182 adult participants that had sufficient DNA, with sufficient RNA recovery from 108 samples (Figure S1). The demographic characteristics of the adult cohort are presented in Table S3.

The ALLIANCE study protocols were approved by the local medical ethics committee of the University of Lübeck (Vote 12–215; 18.12.2012), and all patients or their parent/guardian gave written informed consent. For detailed methods, see the Supporting Information.

The complete inclusion/exclusion criteria of both paediatric and adult arms of the ALLIANCE study were previously described [20].

### 2.4 Ex Vivo Infection of Primary Nasal Epithelial Cells With Human Rhinovirus 16

Nasal DNA methylation data were screened from our in vivo nasal brush collection. Nasal epithelial cell samples from children (*n* = 34 with low and *n* = 19 with high DNA methylation in the *IL6* gene locus) of the paediatric arm of the ALLIANCE cohort were cultured on a six‐well plate precoated with bovine‐derived collagen 1 (C5533, Sigma‐Aldrich, Germany). Cultures were infected with Human Rhinovirus 16 (HRV) at a multiplicity of infection (MOI) of 10 for 48 h at 37°C as previously described [21]. As controls, cells were treated with virus buffer and were designated as MOCK samples.

RNA was isolated from MOCK and HRV‐infected (+HRV) primary nasal epithelial cells using the AllPrep DNA/RNA/Protein Mini Kit (Qiagen, Hilden, Germany). RNA concentration and quality were quantified with the Agilent RNA 6000 Nano Chip Kit (Agilent, Santa Clara, USA). Samples with RNA Integrity Number (RIN) scores greater than 8 were selected for further analysis. Paired‐end RNA sequencing was conducted at the Institute of Clinical Molecular Biology, Christian‐Albrechts‐University of Kiel. rRNA was depleted, and libraries were generated using the TruSeq stranded mRNA Library Prep Kit (Illumina, San Diego, USA) and processed through the NovaSeq 6000 System S4 (Illumina, San Diego, USA) with a read length of 2 × 100 bp. Approximately 25 million reads per sample were obtained.

Subjects were then stratified based on their IL6TS profile, and the association of IL‐6 concentration (ng/ml) was analysed.

### 2.5 Whole Genome Microarray, DNA Methylation and RNA Expression Analysis

Whole‐genome transcriptomic analysis was performed using the SurePrint G3 Human Gene Expression 8 × 60K Microarray, and data were normalised using a global median transformation with log2 fold change computation. DNA methylation analysis was conducted using bisulphite‐converted DNA and the HumanMethylation450 Bead Chip kit, with *β*‐values normalised using Illumina's algorithms. RNA sequencing was performed using the HiSeq2500 System, and reads were aligned using GSNAP, with data expressed as reads per kilobase per million mapped reads (RPKM). Quality control included principal component analysis (PCA) for transcriptomic data and exclusion of compromised microarray signals from further analysis. Further details on the methodologies are provided in the Supporting Information.

## 3 Statistical Analysis

One‐way ANOVA with subsequent Tukey's test was used to determine the significance of differences. Expression and DNA methylation data were analysed using JMP 13 (SAS, USA). Correction for multiple testing was applied where appropriate.

Normality of the data distribution was assessed using the Shapiro–Wilk test. For datasets with fewer than 30 observations or those not following a normal distribution, nonparametric tests (Wilcoxon signed‐rank test, Mann–Whitney U test) were applied. All statistical analyses were conducted using GraphPad Prism (Version 10).

Hierarchical clustering was performed according to Ward's method as implemented in JMP 13. Statistically significant differences were defined as *p < 0.05, **p < 0.01, ***p < 0.001, ****p < 0.0001 and n.s. = nonsignificant. Circular plot representation of IL‐6 trans‐signalling (IL6TS) was generated using ‘*R*’ software (v4.3.0) with the package *circlize* (v0.4.15). *IL6* DNA methylation beta values were feature‐scaled per probe following a mean normalisation method. *IL6* gene DNA methylation, IL‐6 protein production and IL6TS gene signature were aligned in a patient‐specific manner.

## 4 Results

### 4.1 Exacerbation of Experimental Asthma in Mice is Associated With an Exaggerated IL‐6 Release

**FIGURE 1:**
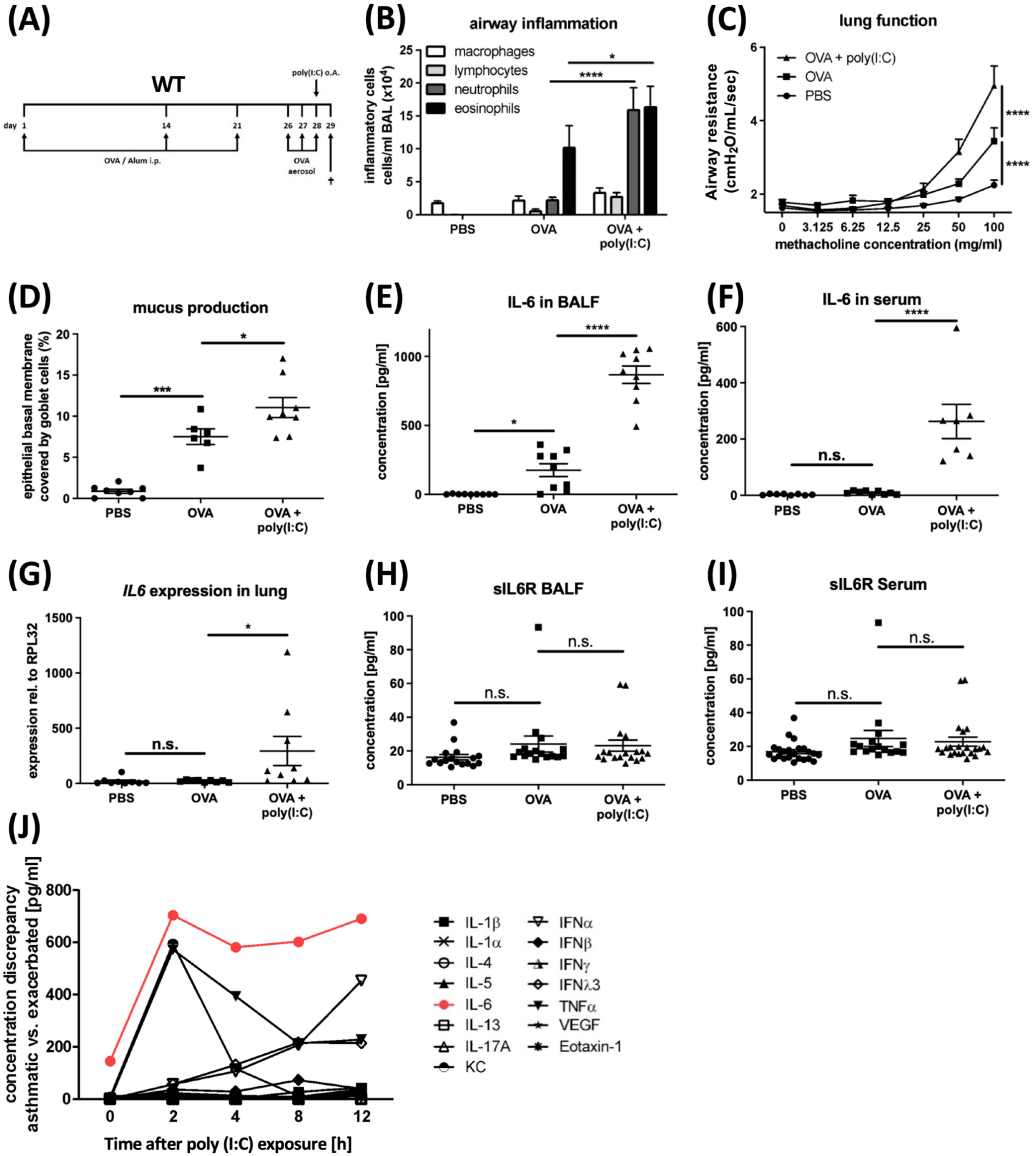
Exacerbation of experimental asthma in mice is associated with an exaggerated IL‐6 response. (A) Treatment protocol of ovalbumin‐induced experimental allergic asthma and poly(I:C)‐triggered exacerbation in wild‐type mice. (B) Numbers of macrophages, lymphocytes, neutrophils and eosinophils in bronchoalveolar lavage fluid (BALF) of PBS (*n* = 8), OVA (*n* = 8) and OVA + poly(I:C) mice (*n* = 8). (C) Airway resistance (cmH_2_O/mL/s) in response to methacholine challenge. (D) Percentage of area of epithelial basal membrane covered by goblet cells. (E) Concentration of IL‐6 in BALF (pg/mL). (F) Concentration of IL‐6 in serum (pg/mL). (G) Expression of *IL6* in lung tissue homogenate. (H) Concentration of soluble IL‐6 receptor (sIL‐6R) in BALF (pg/mL). (I) Concentration of sIL‐6R in serum (pg/mL). (J) Concentration discrepancy of 15 different cytokines (pg/mL) in asthmatic versus exacerbated mice in BALF at five different time points (0, 2, 4, 8 and 12 h) after poly(I:C) exposure.

Local application of poly(I:C), a well‐established surrogate of the viral PAMP (pattern‐associated molecular pattern) dsRNA, induced exacerbation of EAA, which was characterised by aggravated airway inflammation, AHR and mucus production (Figure 1A–D) [22]. Though EAA is already associated with increased levels of IL‐6 in bronchoalveolar lavage fluid (BALF), animals with EAA exacerbation revealed up to four‐fold higher IL‐6 levels in BALF and more than 20‐fold higher serum levels (Figure 1E,F) and with up to 15‐fold higher *IL‐6* expression in lung tissue (Figure 1G), while the release of soluble IL‐6 receptor (sIL‐6R) remained unchanged (Figure 1H,I). Among the 15 analysed mediators previously associated with allergic inflammation or an antiviral immune response (Figure S2A–P), IL‐6 was released in the highest concentration across all time points, reached the earliest recognizable peak and showed the largest response compared to stable disease (Figure 1J).

### 4.2 Acute Exacerbation of Experimental Asthma in Mice Requires an Exaggerated IL‐6 Release

**FIGURE 2:**
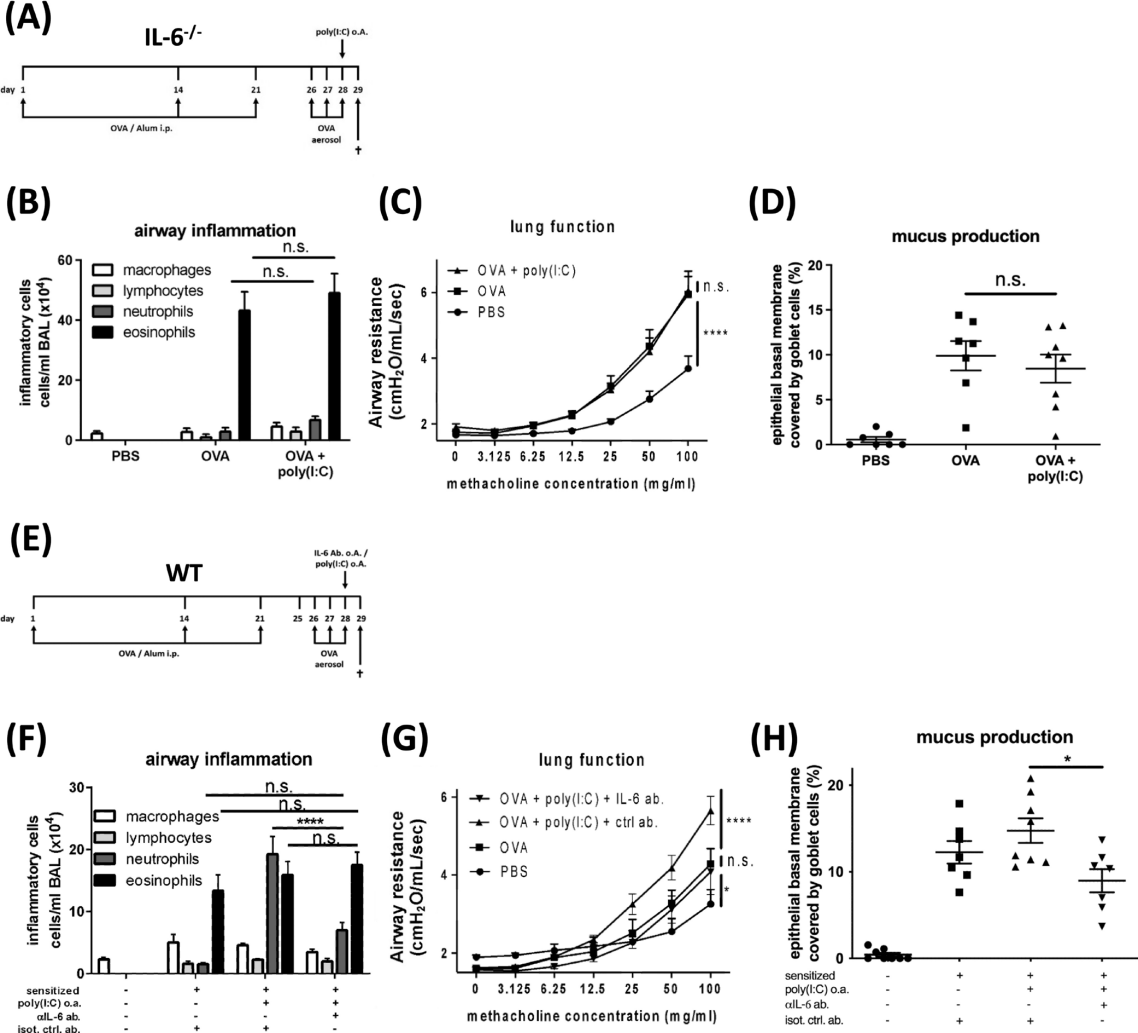
Acute exacerbation of experimental asthma in mice requires IL‐6 release. (A) Treatment protocol of ovalbumin‐induced experimental allergic asthma and poly(I:C)‐triggered exacerbation in IL‐6 knockout mice (B) Numbers of macrophages, lymphocytes, neutrophils and eosinophils in bronchoalveolar lavage fluid (BALF) of PBS (*n* = 8), OVA (*n* = 8) or OVA + poly(I:C) mice (*n* = 8). (C) Airway resistance in response to methacholine challenge. (D) Percentage of area of epithelial basal membrane covered by goblet cells. (E) Treatment protocol of anti‐IL‐6 antibody treatment in an ovalbumin‐induced experimental allergic asthma and Poly (I:C)‐triggered exacerbation in wild‐type mice (F) Numbers of macrophages, lymphocytes, neutrophils and eosinophils in BALF of PBS (*n* = 8), OVA (*n* = 8) and OVA + poly(I:C) mice (*n* = 8) and IL‐6 antibody treated, exacerbated mice (*n* = 8). (G) Airway resistance in response to methacholine challenge. (H) Percentage of area of epithelial basal membrane covered by goblet cells. Results are presented as mean values ± S.E.M. Statistical significance was assessed using ordinary one‐way ANOVA and Tukey's multiple comparison post hoc analyses, *p < 0.05, **p < 0.01, ***p < 0.001 and ****p < 0.0001, n.s., not significant.

Accordingly, in the clinical studies, our animal models displayed an association between exacerbations and increased IL‐6. Thus, we next evaluated the functional relevance of IL‐6 in IL‐6^−/−^ animals. These mice were healthy and displayed the pathophysiological features of EAA, but in contrast to wild‐type animals’ exposure to poly(I:C) did not lead to aggravation of airway inflammation, AHR or mucus production, and thus not to exacerbation (Figure 2A–D). Furthermore, sensitised IL‐6^−/−^ mice revealed higher inflammatory cell counts and mediator levels of, for example, IL‐5 and eotaxin (Figure S3A–I) in BALF than wild‐type animals upon OVA challenge, so that we cannot exclude any effects of IL‐6 on sensitisation or local response to OVA. Therefore, we subsequently pretreated wild‐type animals undergoing the protocol for the induction of poly(I:C)‐induced exacerbation of EAA with an anti‐IL‐6 antibody. Again, neutralisation of IL‐6 prevented aggravation of the pathophysiological features and EAA exacerbation and even lowered neutrophil numbers as compared to animals treated with control antibody (Figure 2E–H; Figure S3J–R), demonstrating the critical role of IL‐6 in the development of EAA exacerbation in mice. Additionally, in mice with EAA, local application of increasing amounts of poly(I:C) resulted in increasing IL‐6 levels in the airways, accompanied by a dose‐dependent aggravation of EAA hallmarks and exacerbation‐like features (Figure S4A–B).

### 4.3 Repeated RV16 Infections Train IL‐6 Release and Induce an IL6TS High Expression Profile in BECs in Vitro

Clinically, previous exacerbations increase the risk of future exacerbations [23]. Thus, we hypothesised that recurrent exposure to respiratory viruses augments subsequent IL‐6 release, eventually by mechanisms involving innate immune training.

IL‐6 production was described for a variety of immune cells as well as for some structural cells, which is in line with immuno‐histochemical staining against CD90.1 of lungs from IL‐6‐reporter mice with acute exacerbation of EAA (Figure S5A–D). FACS analysis did not reveal infiltration of fast‐responding innate immune cells such as γδ T cells, NK cells, NKT cells or ILC cells into the lung (Figure S6A–E). Kinetic studies further revealed that infiltration of neutrophils, another putative source of IL‐6, peaked much later than IL‐6 levels in BALF, and that macrophage numbers did not change over time (Figure S7A,B). Thus, we decided to further focus on airway epithelial cells (AEC), which (beneath endothelial cells) exhibited strong IL‐6 reporter staining (Figure S5A) and are described to act as both sentinel cells to and target cells for respiratory viruses [24].

**FIGURE 3:**
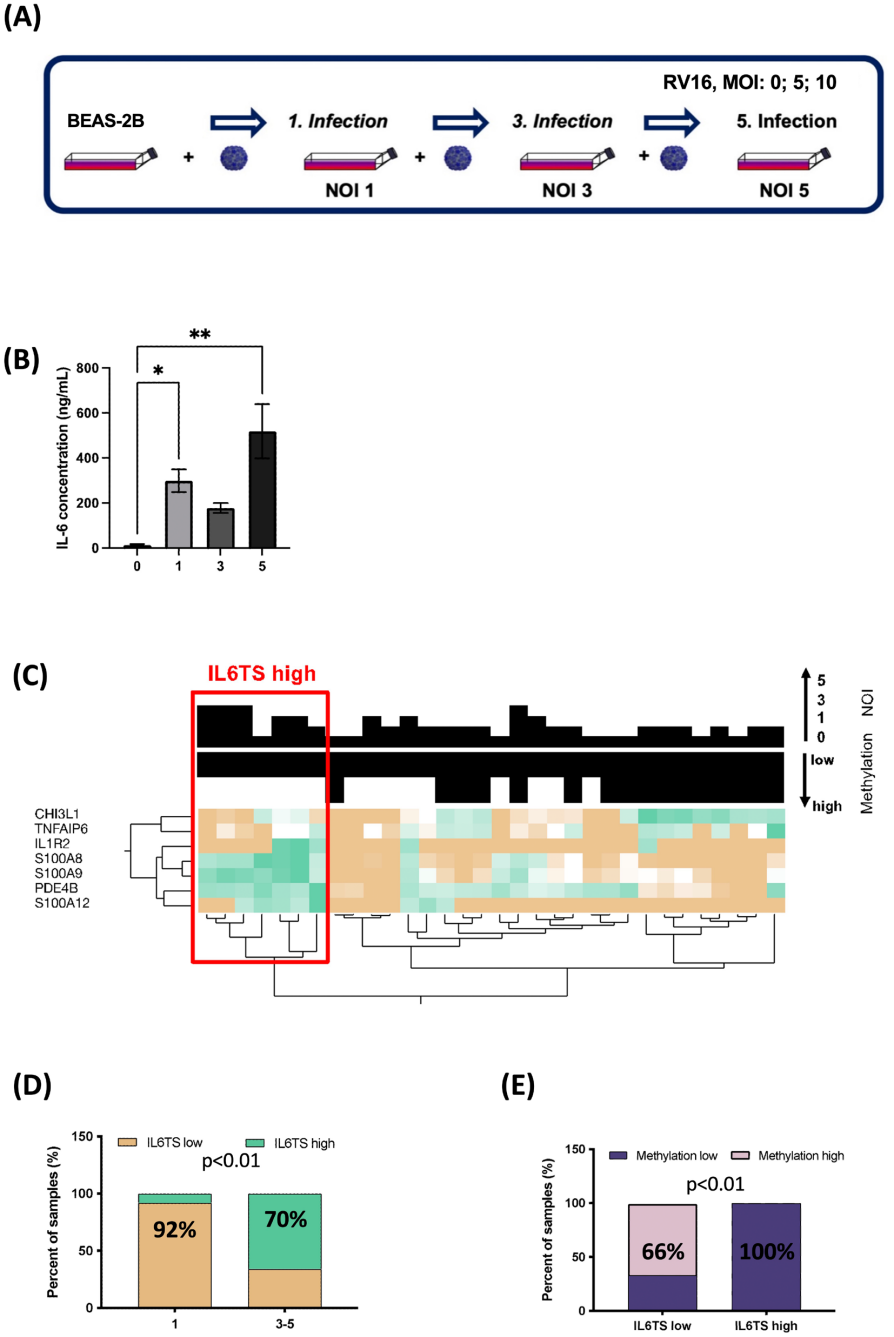
Repeated RV16 infections train release of IL‐6 and induce an IL‐6 trans‐signalling (TS) high expression profile in bronchial epithelial cells in vitro. (A) Repeated infection protocol of BEAS‐2B cells with human rhinovirus‐16 (RV16). (B) IL‐6 concentration in cell culture supernatant after 0, 1, 3 and 5 infections. Results are presented as mean values ± S.E.M. Statistical significance was assessed using ordinary one‐way ANOVA and Tukey's multiple comparison post hoc analyses, *p < 0.05, **p < 0.01, ***p < 0.001 and ****p < 0.0001, n.s. = not significant. (C) Expression profile of IL6TS genes (*CHIL3L1, TNFAIP6, IL1R2, S100A8, S100A9, PDE4B* and *S100A12*) in BEAS‐2B cells after 0, 1, 3 and 5 infections with RV16 depicted as a heat map, divided into an IL6TS high and low profile, and associated with methylation of the *IL6* gene. (D) Percentage of samples with 1 and 3–5 infections that show a low or high IL6TS expression profile. (E) Percentage of samples with low or high IL6TS profiles that show a low or high methylation of the *IL6* gene respectively. Chi‐square test for proportions.

Hence, BEAS‐2B cells were recurrently stimulated in vitro with poly(I:C). *IL6* expression significantly increased with the number of exposures (NOE) (Figure S8A), similar to mice with EAA features that underwent repeated poly(I:C) instillations (Figure S8B). Therefore, to realistically model viral infection, we subsequently infected BEAS‐2B cells repetitively with RV16 (Figure 3A). This resulted in a significant increase in IL‐6 release (Figure 3B). We next interrogated the IL6TS deploying a gene expression profile developed by Jevnikar and colleagues [11]. This profile consists of seven genes (CHIL3L1, TNFAIP6, IL1R2, S100A8, S100A9, PDE4B and S100A12) (Table S4), which we clustered (Ward) for high versus low profile (Figure 3C). In 70% of BEAS‐2B cultures with repeated infections (NOI 3–5, RV16), IL‐6 release led to an IL6TS high profile. Conversely, IL6TS high was minimal (8% of cultures) in single infection experiments (NOI 1) (Figure 3D; Chi‐square: *p* < 0.01). Intriguingly, cells with an IL6TS high profile predominantly showed hypomethylation of the *IL6* gene. Consequently, cells with an IL6TS low profile were hypermethylated at the *IL6* gene (IL6M) (Figure 3E; Chi‐square: *p* < 0.01 and Figure S9). Hence, increased release of IL‐6 in recurrently infected BEAS‐2B's correlated with both hypomethylation of the *IL6* gene and an IL6TS high profile. These data suggest that recurrent viral infections trained the increased epithelial IL‐6 release.

### 4.4 Hypomethylation of the *IL6* Gene is Associated With Increased *IL6* Expression, Release and a High IL6TS Expression Profile In Vitro and in Asthmatic Patients

**FIGURE 4:**
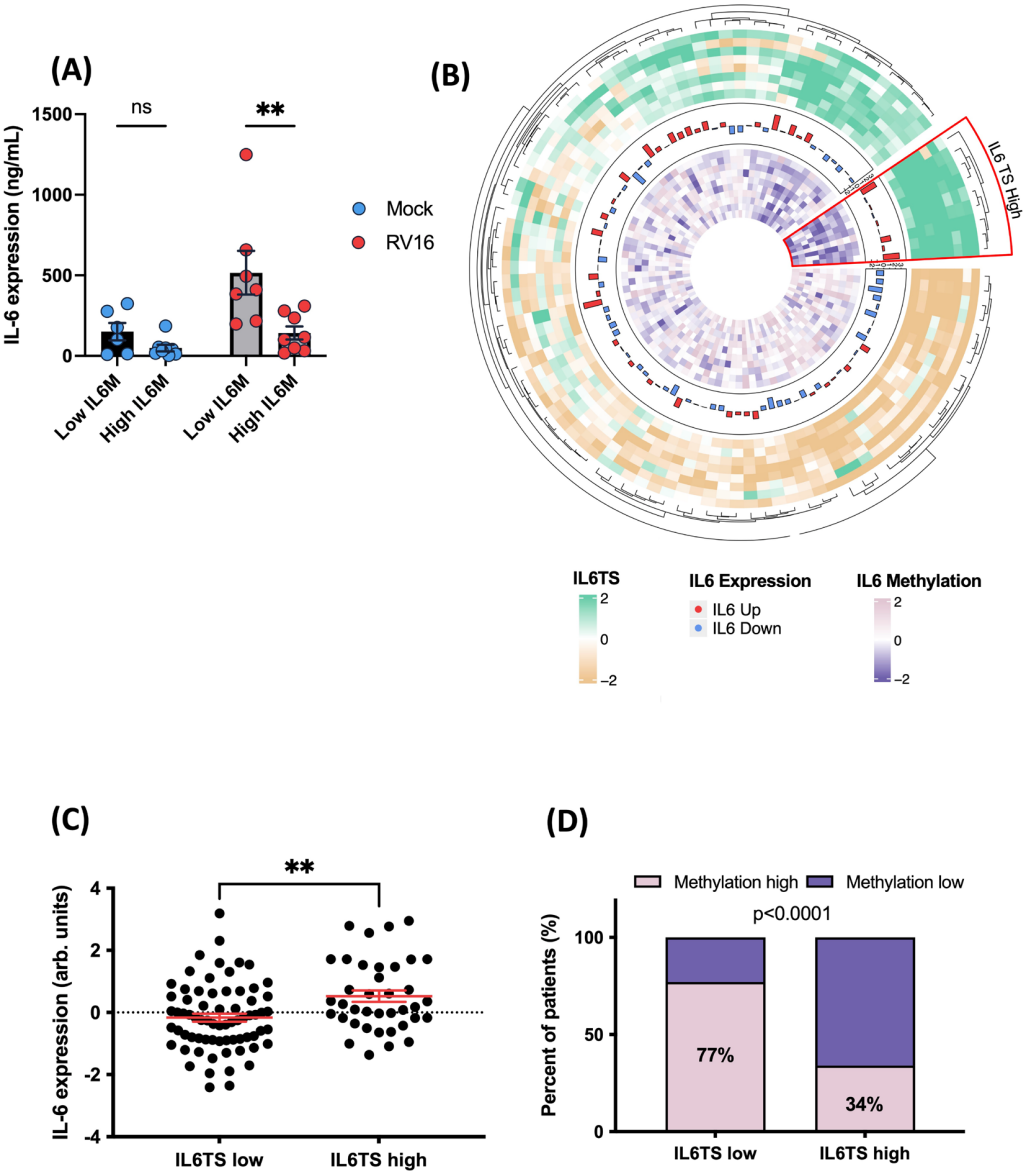
Hypomethylation of the *IL6* gene is associated with increased IL6 release, a high IL6TS expression profile and an increased IL6 expression in vitro and in asthma patients. (A) IL‐6 expression in human primary nasal epithelial cells, stratified for low and high IL‐6 methylation (IL6M), infected by either mock (blue) or human RV‐16 (RV16) (red) (*p* < 0.01). Results are presented as mean values ± S.E.M. Statistical significance was assessed using one‐way ANOVA and Sidak's multiple comparison post hoc analyses, **p < 0.01, and n.s. = not significant. (B) Circular representation of the relationship between IL6TS (outside heat map), IL6 expression (middle bar plot) and IL‐6 DNA methylation (inside heat map). The red segment highlights the IL6TS high cluster of asthmatic patients associated with high IL6 expression and high DNA methylation levels. All rings and the circular plot are coloured according to the legend. (C) IL‐6 expression in asthmatic patients with high (*n* = 38) and low IL6TS (*n* = 70). Results are presented as mean values ± S.E.M. Statistical significance was determined by a Mann–Whitney test, **p < 0.01, n.s. = not significant. (D) Percentage of samples with low or high IL6TS profiles that show a low or high methylation of the *IL6* gene respectively. Chi‐square test for proportions.

To link low IL6M to increased IL‐6 protein release in human respiratory epithelium, we infected primary nasal epithelial cells (NEC) with RV16, comparing low and high IL6M groups in the paediatric arm of the ALLIANCE cohort. Significantly more IL‐6 protein was released after RV16 infection in cells with low IL6M as compared to high (*p* < 0.01, Figure 4A). Similarly, we cultured primary bronchial epithelial cells from asthmatics at air–liquid interface (ALI), stratified by IL6M low (cg15703690 DNA methylation < 50th percentile) or high (cg15703690 DNA methylation ≥ 50th percentile, Figure S10A). Poly(I:C) stimulation led to a significant *IL6* expression increase (*p* < 0.05) in IL6M low versus IL6M high cells. IL6M effect persisted in an IL‐13 stimulated tissue culture mimicking T2 high‐like conditions (low vs. high, *p* < 0.05, Figure S10A).

To translate this fundamental mechanism from children to adults, we then analysed NECs from the adult arm of the ALLIANCE cohort (Table S3) and explored the association of IL6M low, increased IL‐6 expression and a high IL6TS profile in asthmatic patients. First, we clustered the patients according to the IL6TS profile (outer ring Figure 4B, Table S5) and subsequently compared *IL6* gene expression (circular tower plot Figure 45B, Table S6) and methylation (inner ring Figure 4B, Table S7) to each other. First, an IL6TS high expression profile had significantly higher IL6 expression (Figure 4C). Second, low IL6M (67% of patients) coincided with the IL6TS high profile (Chi‐square *p* < 0.0001, Figure 4D). We then ascertained the strong linkage of IL6M and IL6TS in another independent cohort previously reported by Nicodemus‐Johnson et al. (cohort I^18^ details please see Table S2).

Subjects were clustered according to the IL6TS profile genes in Cohort I (Figure S10B). Next, IL6M was subdivided, revealing two distinct subgroups in Cohort I (Figure S11A). Low IL6M significantly coincided with the IL6TS high profile (Chi‐square *p* < 0.05, Figure S11B). Predominantly, methylation at cg15703690 and cg01770232 was reduced in the IL6TS high expression profile group (*p* < 0.01, Figure S11C–F).

Taken together, the in vitro and ex vivo data provide strong evidence of a correlation between low IL6M and high *IL6* gene expression and IL‐6 protein release in human NECs and BECs. In addition, IL6TS high expression profiles in the nose and bronchus were associated with low IL6M in patients with asthma.

### 4.5 Low *IL6* Gene Methylation in Respiratory Epithelial Cells of Patients With Asthma Occurs in T2 High or Low Endotypes and is Linked to Previous Exacerbations

**FIGURE 5:**
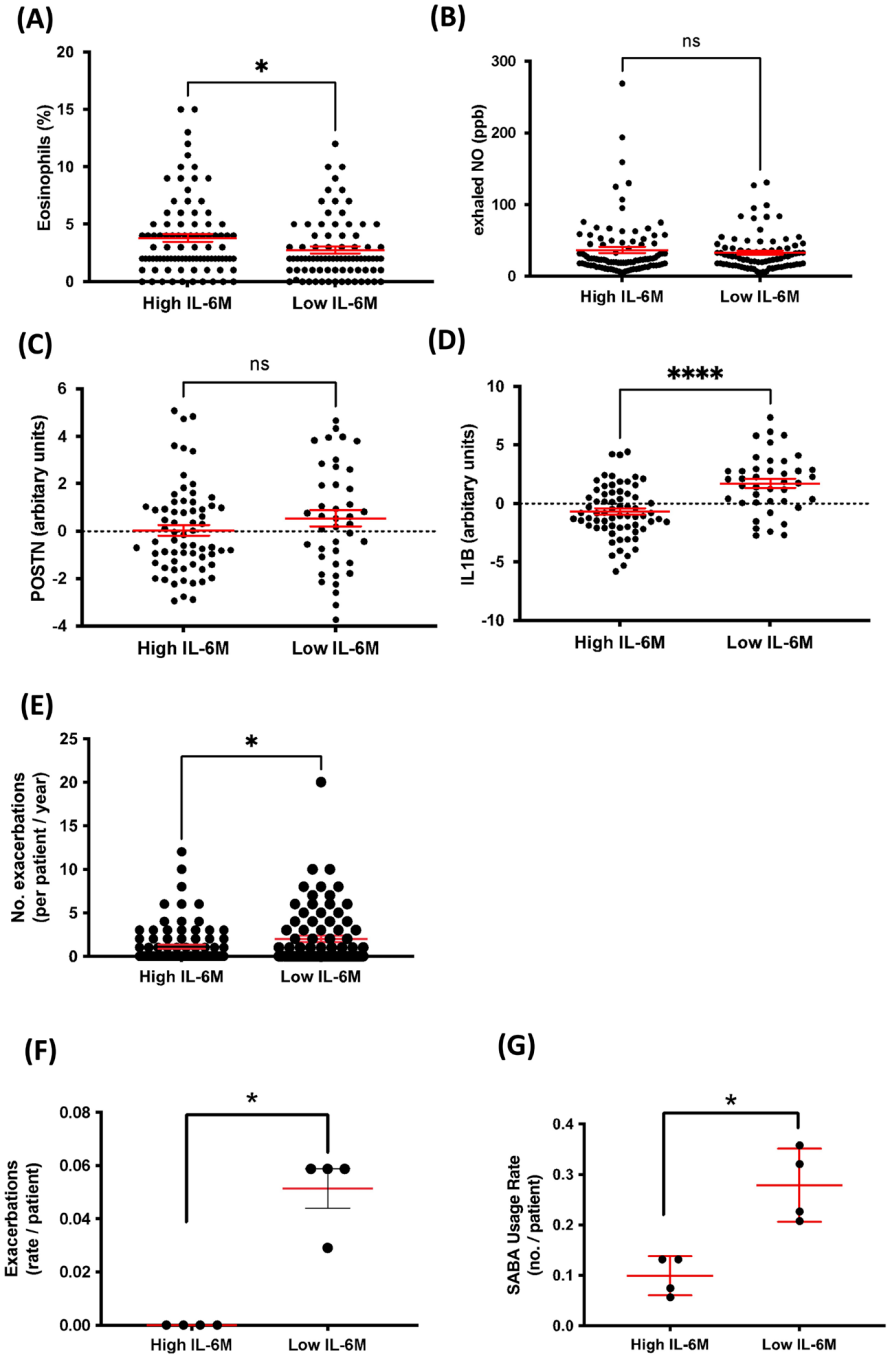
Low *IL6* gene methylation in respiratory epithelial cells is independent of the T2 endotype in the adult arm of the ALLIANCE cohort and associated with future exacerbations and rescue medication use in asthmatic children. (A) Blood eosinophil percentages in patients with asthma who have a high and low *IL6* gene methylation. (B) Exhaled nitrogen oxide in patients with asthma who have a high and low *IL6* gene methylation. (C, D) Periostin and IL1B gene expression compared between high and low *IL6* gene methylation levels. (E) Exacerbation rate per patient with high and low *IL6* gene methylation levels. Results are presented as mean values ± S.E.M. Statistical significance was assessed using Mann–Whitney test *p < 0.05, ****p < 0.0001, and n.s., nonsignificant. All data are from the adult arm of the ALLIANCE cohort. (F) Mean exacerbation rate per patient group in paediatric ALLIANCE patients with high (*n* = 19) and low (*n* = 34) *IL6* gene methylation, where each data point represents multiple patients within the respective groups. (G) Mean short‐acting β‐agonist (SABA, rescue medication) use rate at each visit in paediatric ALLIANCE patients with high (*n* = 19) and low (*n* = 34) IL6 gene methylation. Each data point represents the mean exacerbation rate and mean SABA use rate for all patients in the respective group. Visits include baseline and three consecutive yearly follow‐ups. Results are presented as mean values ± S.E.M. Statistical significance was assessed using the Mann–Whitney test *p < 0.05.

IL6TS high patients were previously shown to have elevated blood eosinophil levels that were not linked to epithelial T2 high signatures [11]. Patients in the adult arm of the ALLIANCE cohort with low IL6M had a reduced blood eosinophil percentage (*p* < 0.05, Figure 5A) and were older (*p* < 0.05, Table S3) than the IL6M high group, with no significant differences in exhaled NO (Figure 5B), BMI or FEV_1_% predicted (Table S3).

In contrast, patients in Cohort I with low IL6M had a higher blood eosinophil percentage (*p* < 0.05, Figure S12A) and lower exhaled NO (*p* < 0.01, Table S4) than the IL6M high group, with no significant differences in age, height, weight, BMI or FEV_1_% predicted (Table S4).

Of note, STEP treatment classes [25] tended (n.s.) to be higher in the lower IL6M clusters, consistent with an association between low IL6M and increasing asthma severity in Cohort I (Table S2).

Next, we analysed expression of the periostin (*POSTN*) gene, a known signature in BECs for T2‐high asthma [26]. POSTN expression was not significantly different in patients with low IL6M in the adult arm of the ALLIANCE cohort (Figure 5) but increased in patients with low IL6M in Cohort I (*p* < 0.05, Figure S12B). Adult ALLIANCE patients with low IL6M had features of neutrophilic asthma as signified by elevated peripheral neutrophil counts (*p* < 0.05, Table S3) and high interleukin 1b (IL1B) expression (*p* < 0.0001, Figure 5D). No such associations were seen in Cohort I (n.s., Figure S12C). Consequently, we found ALLIANCE patients with low IL6M to exacerbate more frequently 12 months prior to the study visit (*p* < 0.05, Figure 5E). These combined data suggest that an IL6M low phenotype may occur in T2 high or T2 low endotypes and is associated with a more severe phenotype.

### 4.6 Hypomethylation of the *IL6* Gene in Respiratory Epithelial Cells of Paediatric Patients With Asthma is Associated With Future Exacerbations

Patients with asthma with high IL6TS have previously been reported to experience more exacerbations [11]. After linking high IL6TS to low IL6M, we subsequently assessed the effects of low IL6M in NECs on future exacerbations in children from the ALLIANCE for three consecutive yearly follow‐up (FU) visits (Table 1). Paediatric asthmatics were clustered into high versus low IL6M (Figure S13A). The proportion of high IL6M was increased in the low IL6TS versus high (Chi‐square *p* < 0.05, Figure S13B,C).

Overall, children with high IL6M had similar levels of atopy and ICS use, reported a similar level of GINA symptom control at baseline, and similar total serum IgE, eosinophil blood concentration and lung function parameters compared to children with low IL6M (Table 1). Children with high IL6M were more likely to be male and were significantly older at baseline compared to low IL6M (*p* < 0.05).

During follow‐up, there were overall few differences between the low and high IL6M low and high children, except for the number of exacerbations (Table 1). There was a trend to improved FEV_1_ in the high IL6M but not in the low IL6M group. Furthermore, the blood eosinophil counts significantly increased over time in the low IL6M subgroup, while decreasing in the high IL6M (*p* < 0.01 at FU3). Across four visits, the mean frequency of patients with exacerbations (as defined by the GINA questionnaire) was significantly (*p* < 0.05) increased in low IL6M (3.34%, *n* = 34 patients) compared to high IL6M (0%, *n* = 19, Figure 5F). Supporting the notion of more aggravated disease, in the low IL6M group, the mean rate (over four visits) of emergency reliever use (as defined by the GINA questionnaire) was significantly increased in the low compared to the high IL6M group (27.85% vs. 9.45%, *p* < 0.05; Figure 5G). In addition, ICS use significantly decreased over follow‐up in high IL6M compared to the low IL6M group (Table 1), without any changes to the level of GINA control. These findings suggest that children with asthma who also have low IL6M in NECs are at higher exacerbation risk, have elevated levels of blood eosinophils, use more reliever medication, and are less likely to achieve asthma control.

## 5 Discussion

In the present study, we found that poly(I:C)‐induced exacerbation of experimental asthma in mice depends on IL‐6 release, and that repeated exposure to poly(I:C) leads to increased IL‐6 release by BECs and increased airway inflammation. Furthermore, exaggerated IL‐6 release in human BECs results from innate immune training by repetitive infection with RV16 (or poly(I:C)), that is, in turn, associated with IL6TS high expression and a low *IL6* gene methylation profile. Finally, in NECs of asthmatic patients, low *IL6* gene methylation is also associated with an IL6TS high expression profile and increased *IL6* expression, and with future exacerbations in children.

Elevated levels of IL‐6 and IL‐6TS in asthmatic patients have been associated with more severe disease across multiple patient subsets, and moreover, experimental exposure has shown that IL‐6 and IL‐6TS components are upregulated in the airways, suggesting that stimulus‐induced IL‐6 release may further contribute to airway inflammation and disease severity [27, 28], implicating IL‐6 in the pathogenesis and exacerbations of asthma. Consistent with previous studies, we report elevated production of IL‐6 in animal lungs with EAA [22, 29]. Mediator release, mucus production or AHR were unaltered in IL‐6^−/−^ animals; however, inflammatory cell counts and levels of IL‐5 and eotaxin in BALF are higher than in wild‐type animals, suggesting a nuanced role of IL‐6 in disease formation. Instillation of poly(I:C) to this OVA‐induced, experimental asthma model elicited an immediate and profound IL‐6 response and aggravated the allergic airway inflammation. While IL‐6 release preceded all other measured mediators, no change in the release of sIL‐6R was noticeable. We demonstrated its central importance by the abrogation of the aggravating effects of poly(I:C) in IL‐6‐deficient animals and a proof‐of‐concept treatment with an IL‐6‐neutralising antibody prior to instillation. These findings indicate that IL‐6 is critically required for the development of poly(I:C)‐induced exacerbation. The exact mechanistic role of IL‐6 remains to be elucidated.

Our immune‐histological staining underpins the primacy of the airway epithelium for substantial release of IL‐6 after poly(I:C) stimulation. This, in turn, may arise from activation of pattern recognition receptors (PRRs) such as TLR‐3 (Toll‐like Receptor 3), RIG‐I (retinoic acid inducible gene I) and MDA‐5 (melanoma differentiation‐associated protein 5) comparable to viral double‐stranded RNA [30], and parallel or affect antiviral innate immune responses (e.g., type I/III interferons) as it has been reported previously as possible mechanisms to contribute to asthma exacerbations. Hence, IL‐6 directly and/or indirectly promoted the existing allergic inflammation, thereby worsening the pathophysiological hallmarks of experimental asthma. Other hyperinflammatory conditions in the lung such as acute respiratory distress syndrome (ARDS) and coronavirus disease‐2019 (COVID‐19) are also often associated with elevated IL‐6 production [31, 32]. Hence, our findings suggest IL‐6 as a putative target for the prevention and therapeutic intervention of such acute, severe disease states.

Though the cellular source of IL‐6 in asthma exacerbations remains uncertain, it is known to be produced by innate immune cells (e.g., macrophages, neutrophils, dendritic cells), T cells and structural cells (e.g., epithelial or endothelial cells) [33]. We decided to further focus on AECs due to several aspects: (I) Recent studies highlighted the bronchial epithelium as a major contributor to IL‐6 production in asthma patients and in experimental models, suggesting its significance in exacerbations [34, 35]. (II) AECs could also be trained by microbial stimuli [36]. (III) AECs serve as the first point of contact for external stimuli such as respiratory viruses, acting as both primary target and immune sentinels; thus, holding a crucial position in initiating immune responses against any pathogens [24]. (IV) Kinetic studies suggested AECs to be the earliest producers of IL‐6 in our animal model.

Frequent exacerbations are reported to be causally linked to viral exposure [37]. We, therefore, hypothesised that exaggerated IL‐6 release is not necessarily caused by the trigger alone but rather may result from the host response to the trigger. Indeed, we found that repetitive RV16 infections led to reduced *IL6* gene methylation in airway epithelial cells, which resulted in increased *IL6* expression, release and subsequently activated an IL6TS. We were able to replicate this fundamental training response in vitro and in vivo by repetitive stimulation of PRRs by poly(I:C) alone. This may suggest that an exaggerated IL‐6 response could be the result of innate immune training.

Our analyses of nasal and bronchial biopsies from patients with asthma established a clear correlation between elevated *IL6* gene expression, IL6TS signatures and *IL6* gene methylation. Changes to *IL6* gene methylation have been shown to be regulatory for gene expression and downstream signalling, such as the IL6TS signature [38]. Given that not only training but also locality (i.e., respiratory epithelium) is key for an augmented IL‐6 response, we hypothesised that lower *IL6* gene methylation would be an indicative of acute and future exacerbations or higher symptom burden. Indeed, adult asthma patients with low IL6M experienced twice the number of exacerbations, and in our paediatric asthma cohort, low IL6M in nasal brushes was associated with future exacerbations. Additionally, among children in the low IL6M group, mean blood eosinophil count peaked at 595 cells/μL, and twice the number of children had to use ICS as compared to the high IL6M group during the follow‐up period. The highest persistence rate in paediatric asthma in ALLIANCE was linked to a T2 high phenotype (> 360 per μL eosinophils and a sensitisation ≥ 07 kU/L) [39]. This suggests that, against the background of allergic asthma, maladaptive immune training of the respiratory epithelium during childhood eventuates in a highly persistent phenotype possibly extending to adulthood.

Thus, we propose a distinct sub‐endotype with a directly actionable therapeutic trait (i.e., inhibition of IL‐6 or IL‐6R). Such antibodies are already approved for the treatment of inflammatory diseases [40], and anti‐IL‐6R therapy may be effective in patients with severe, persistent, steroid‐resistant asthma [41]. However, prospective, longitudinal studies are required to better understand the maladaptive epithelial training and its potential therapeutic interventions.

Our study is limited by a small number of individuals with low IL6M or IL6TS. The nasal brushes from the adult ALLIANCE cohort had sufficient overall size to correlate DNA‐methylation and gene expression, yet an enrichment for low IL6M or increased IL6TS would have been optimal. We overcame this limitation by using several independent HBEC tissue culture independent cohorts (bronchial biopsies and nasal brushes), all showing similar correlations between the DNA‐methylation level and gene expression or trans‐signalling. Secondly, patients in the paediatric cohort were required to be free of symptoms of infections for 14 days prior to any visit [20], limiting the conclusions that can be drawn on acute, exacerbation‐associated cytokines. Yet, the nasal mucosa is highly discriminative for asthma phenotypes in children [42]. Despite a pilot‐study‐size dataset, we successfully used a longitudinal approach to link low IL6M with future exacerbations. Of course, larger prospective and confirmatory studies are warranted to overcome this limitation in our current study design. Lastly, our mechanistic understanding of trained immunity relies on a repeated infections in vitro model. Despite RV16 being carefully removed prior to sub‐cultivation, we cannot fully rule out the possibility of minimal RV16. However, we did not detect residual RV16 transcript reads and IL6 protein levels remained very low in Mock controls, arguing against residual viral activity (Table S1 & 2).

In conclusion, an exaggerated IL‐6 release is required for exacerbation of experimental asthma, resultant of viral PAMP‐induced innate immune respiratory epithelium training. Additionally, patients with asthma and with trained IL‐6 response, defined by hypomethylation of the *IL6* gene, exacerbated more frequently, with paediatric patients showing features of a more severe disease course in the future. These findings may open new avenues to identify and treat exacerbation‐prone asthma patients.

## Author Contributions

L.P.L., Ma.W. and Mi.W. designed the experiments, performed the analysis and wrote the manuscript. L.P.L., Mi.W., R.B.‐S., S.W., C.V. and C.G. planned and performed the mouse models. C.V. and J.E. designed and performed the experiments with ALI cell cultures. C.G. designed and performed the sIL‐6R ELISA. C.B.S.W., C.J. and U.M.Z. designed and performed RNA extraction, gene expression analysis and data analysis of nasal epithelial cells from nasal brushings. C.O. provided data from cohort study I and edited the manuscript. L.P.L., Ma.W., A.Ö.Y., E.V.M., K.D.R. and Mi.W. extensively reviewed and edited the manuscript. B.G.G.O., R.F.C., S.S.P.N. and Ma.W. designed and performed the in vitro infection experiments. Ma.W., K.D.R., A.K., H.B., C.V. and M.v.d.b. Analysed the methylation, expression and trans‐signalling data analysed of the cohorts and edited the manuscript. I.K. analysed data, designed statistical approaches and edited the manuscript. T.B., A.‐M.D., B.S., G.H., K.D.R., E.V.M., G.H. and M.K. designed the ALLIANCE cohort recruitment scheme, recruited participants, collected specimens and edited the manuscript. All authors approved the final version of the manuscript.

## Conflicts of Interest

The authors declare no conflicts of interest.

## Supporting information


**Data S1:** all70070‐sup‐0001‐Supinfo.zip.

## Data Availability

The data that support the findings of this study are available on request from the corresponding author. The data are not publicly available due to privacy or ethical restrictions.
