# Supplementary material for: Immune Training of the Interleukin 6 Gene in Airway Epithelial Cells is Central to Asthma Exacerbations
Source: Allergy. 2025 Oct 16;81(1):157–69. doi: 10.1111/all.70070 (PMC12773691; doi:10.1111/all.70070)
Supplement: Supplementary file 1 — Data S1: all70070‐sup‐0001‐Supinfo.zip. [file ALL-81-157-s001.zip › all70070-sup-0001-FigureS6-S20250513@Lunding et al. IL-6 suppl. figures revised and clean - 20250513.pptx]

## Slide 1
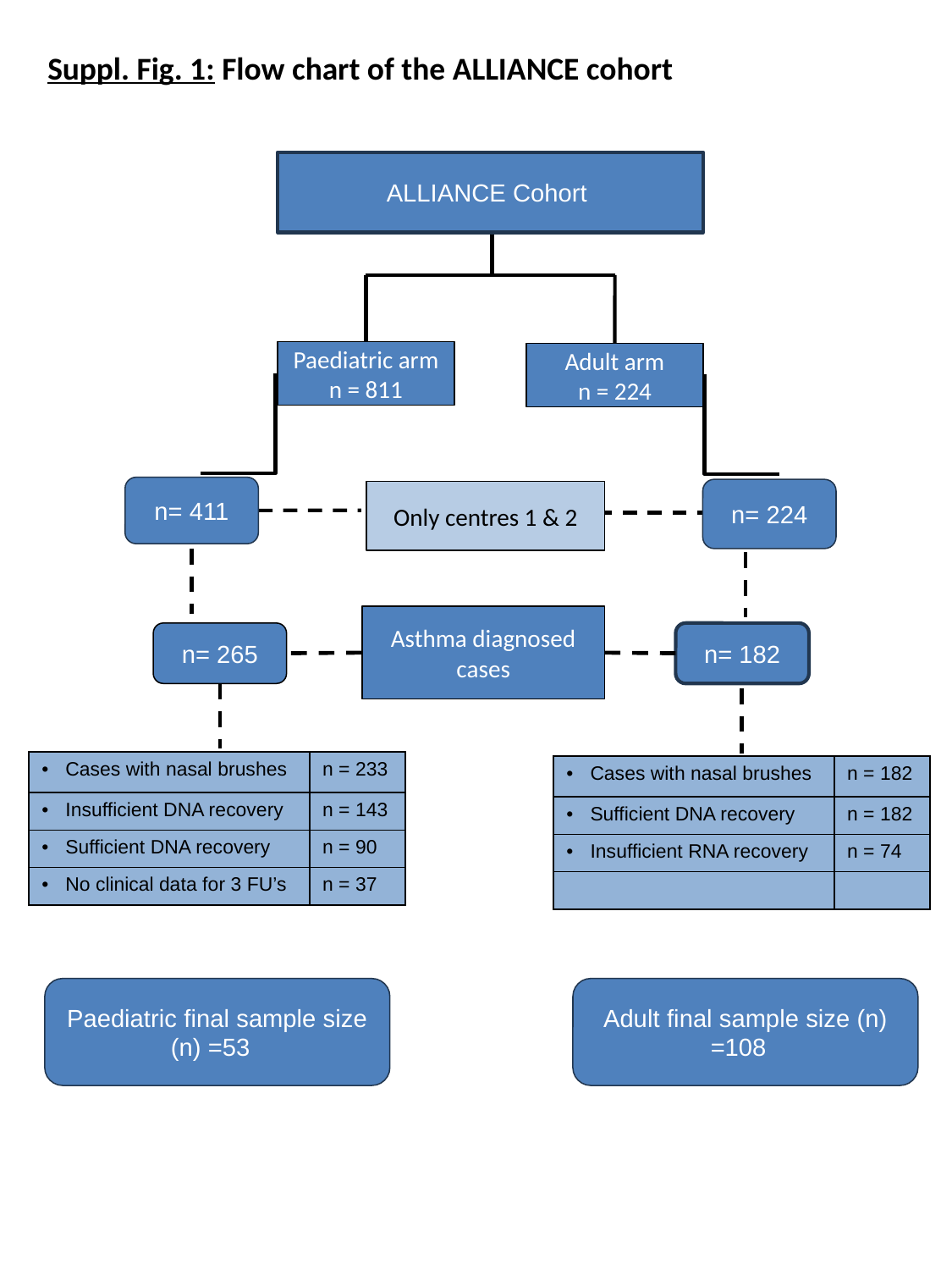

Suppl. Fig. 1: Flow chart of the ALLIANCE cohort
ALLIANCE Cohort
Paediatric arm
n = 811
Adult arm
n = 224
n= 411
n= 224
Only centres 1 & 2
Asthma diagnosed cases
n= 265
n= 182
| Cases with nasal brushes | n = 233 |
| --- | --- |
| Insufficient DNA recovery | n = 143 |
| Sufficient DNA recovery | n = 90 |
| No clinical data for 3 FU’s | n = 37 |
| Cases with nasal brushes | n = 182 |
| --- | --- |
| Sufficient DNA recovery | n = 182 |
| Insufficient RNA recovery | n = 74 |
| | |
Adult final sample size (n) =108
Paediatric final sample size (n) =53

## Slide 2
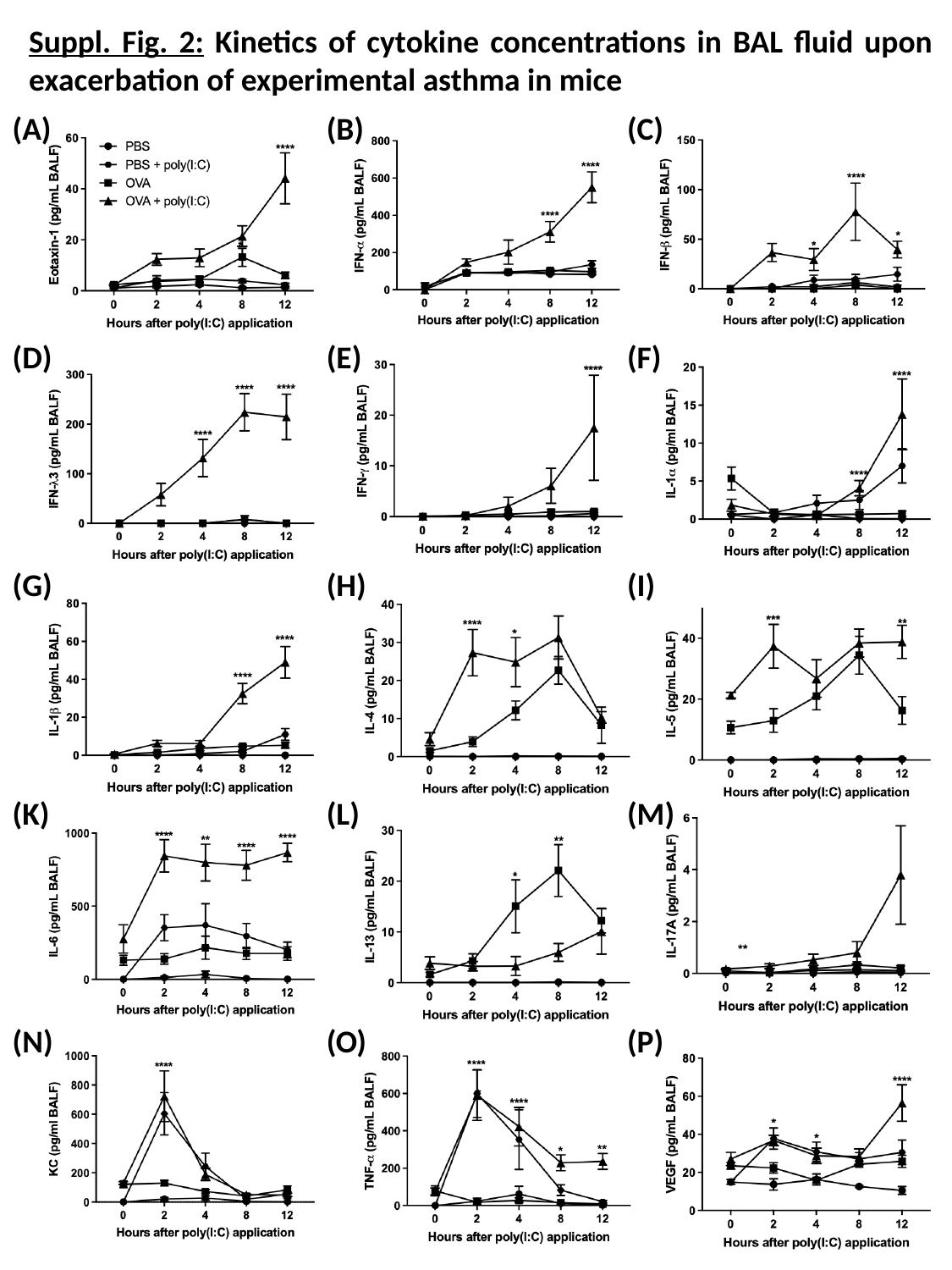

Suppl. Fig. 2: Kinetics of cytokine concentrations in BAL fluid upon exacerbation of experimental asthma in mice
(A)
(B)
(C)
(D)
(E)
(F)
(G)
(H)
(I)
(K)
(L)
(M)
(N)
(O)
(P)

## Slide 3
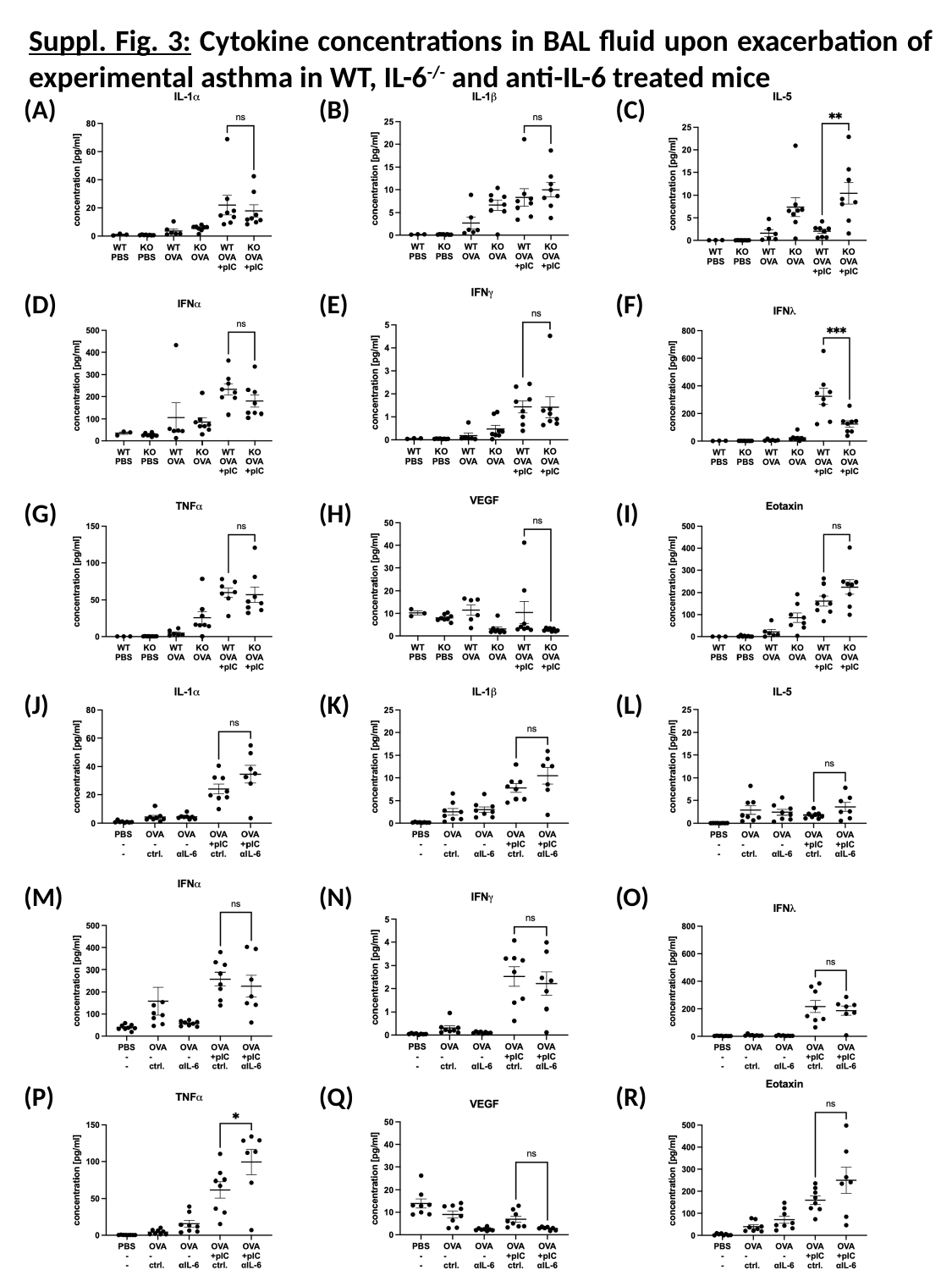

Suppl. Fig. 3: Cytokine concentrations in BAL fluid upon exacerbation of experimental asthma in WT, IL-6-/- and anti-IL-6 treated mice
(A)
(B)
(C)
(D)
(E)
(F)
(G)
(H)
(I)
(J)
(K)
(L)
(M)
(N)
(O)
(P)
(Q)
(R)

## Slide 4
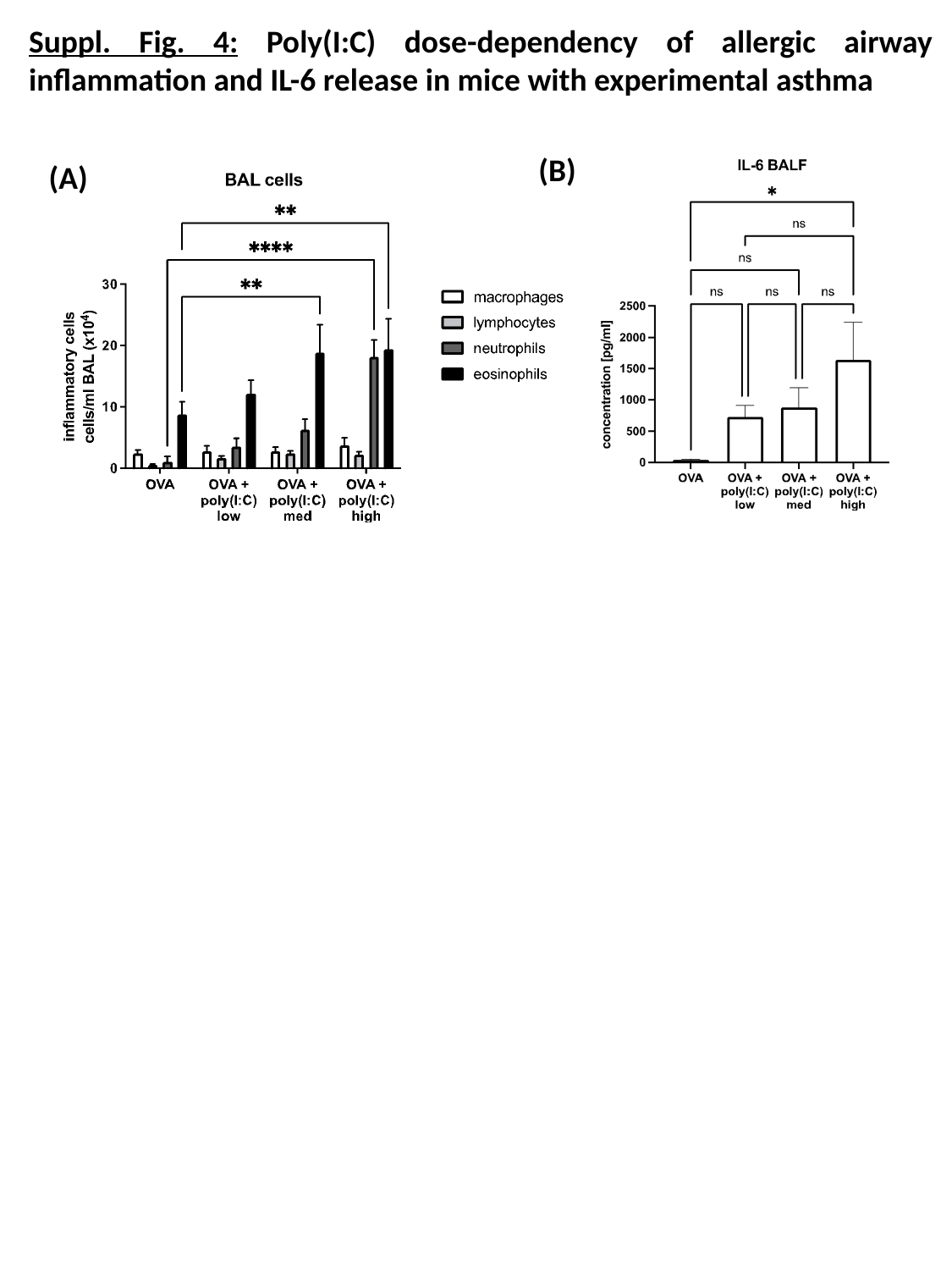

Suppl. Fig. 4: Poly(I:C) dose-dependency of allergic airway inflammation and IL-6 release in mice with experimental asthma
(B)
(A)

## Slide 5
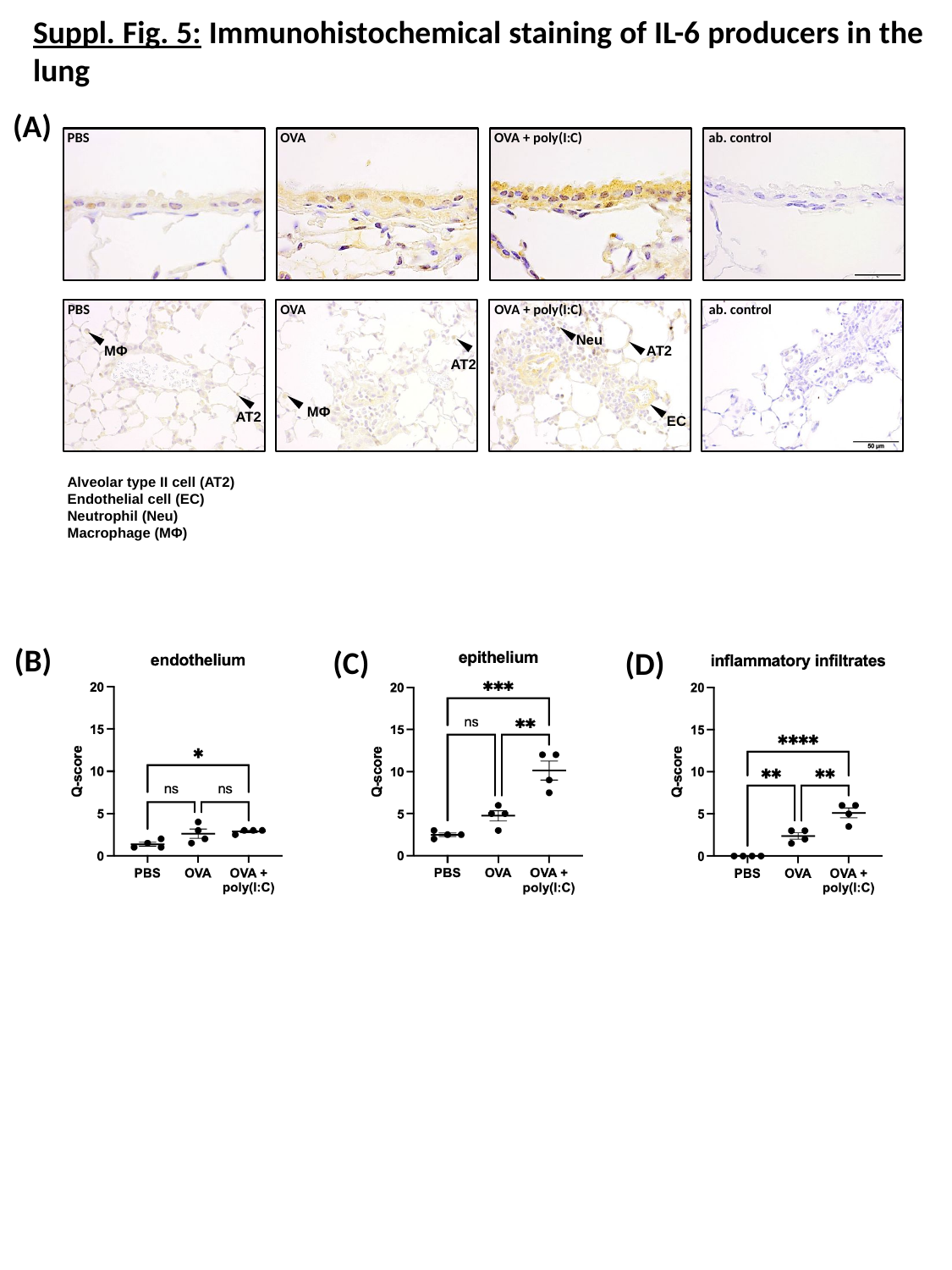

Suppl. Fig. 5: Immunohistochemical staining of IL-6 producers in the lung
(A)
PBS
OVA
OVA + poly(I:C)
ab. control
PBS
OVA
OVA + poly(I:C)
ab. control
Neu
AT2
MΦ
AT2
MΦ
AT2
EC
Alveolar type II cell (AT2)
Endothelial cell (EC)
Neutrophil (Neu)
Macrophage (MΦ)
(B)
(C)
(D)

## Slide 6
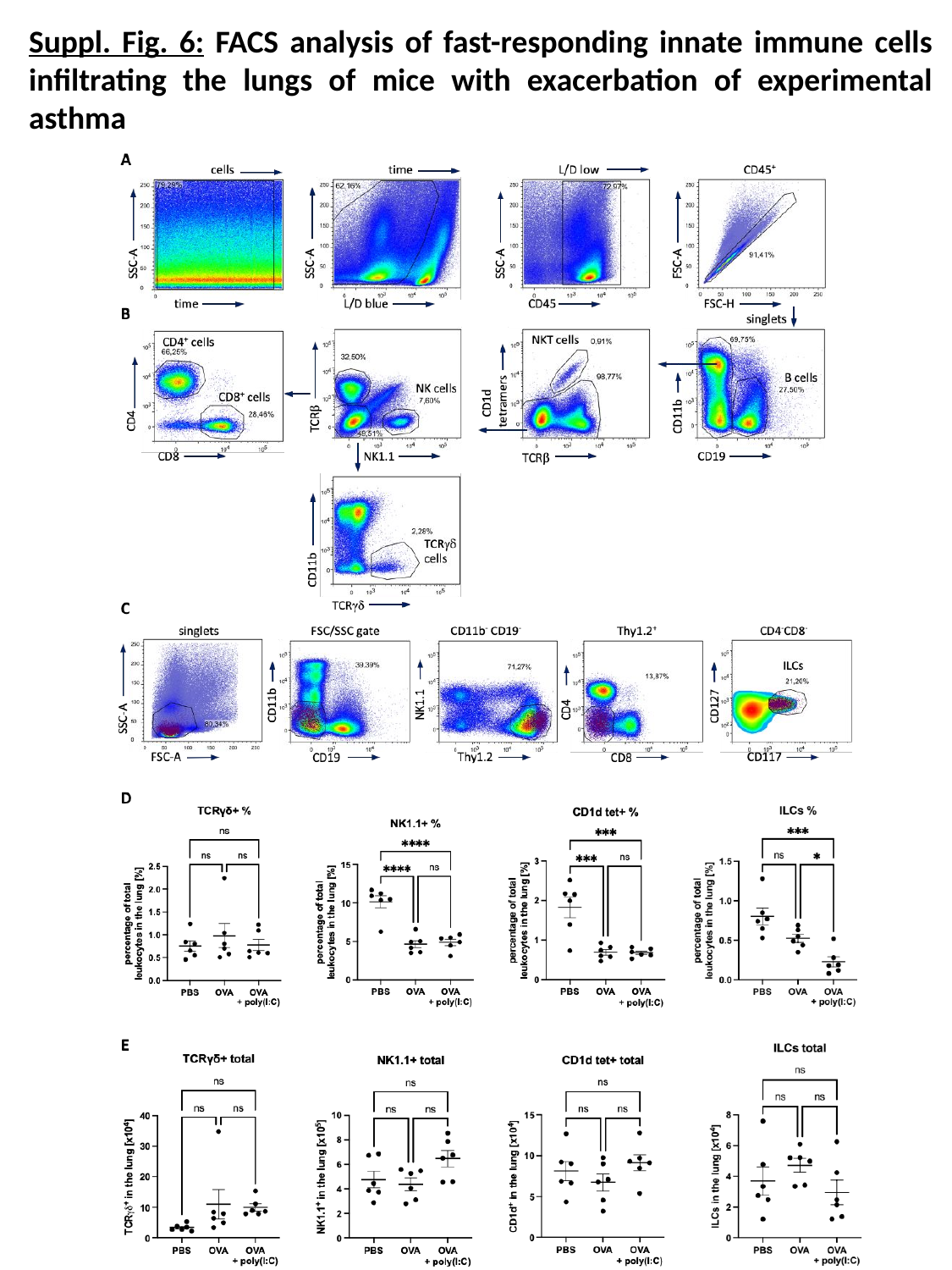

Suppl. Fig. 6: FACS analysis of fast-responding innate immune cells infiltrating the lungs of mice with exacerbation of experimental asthma

## Slide 7
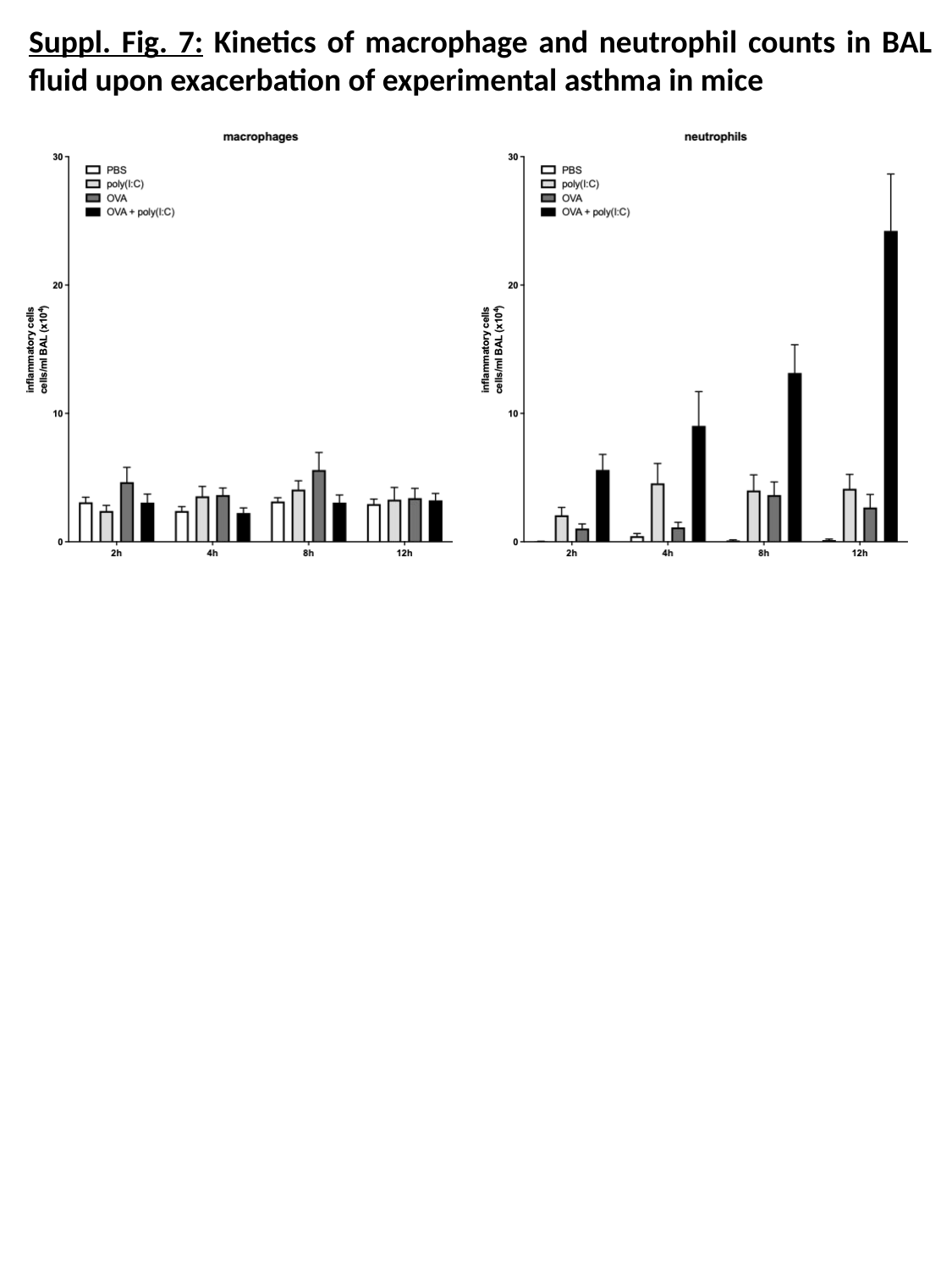

Suppl. Fig. 7: Kinetics of macrophage and neutrophil counts in BAL fluid upon exacerbation of experimental asthma in mice

## Slide 8
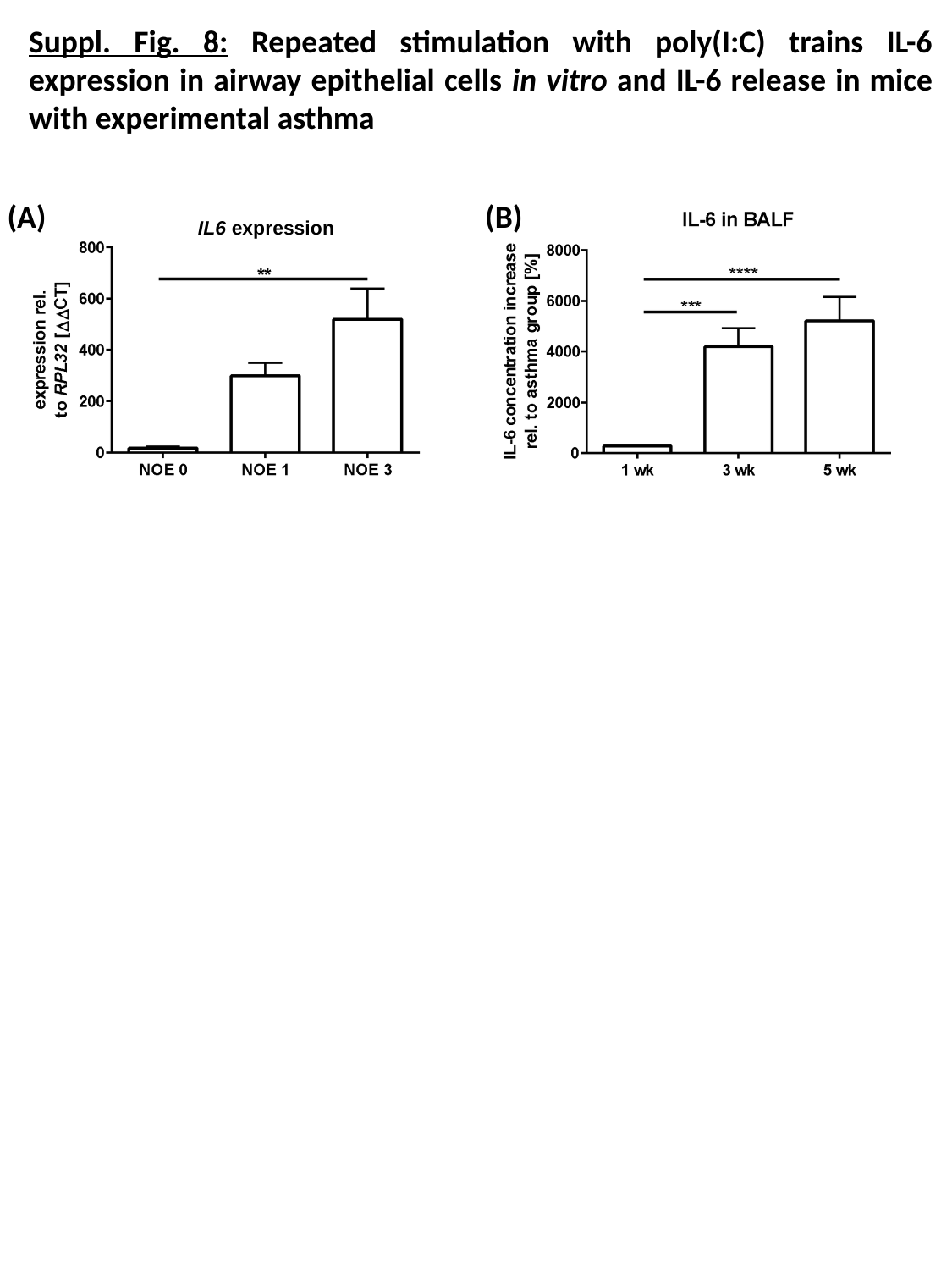

Suppl. Fig. 8: Repeated stimulation with poly(I:C) trains IL-6 expression in airway epithelial cells in vitro and IL-6 release in mice with experimental asthma
(A)
(B)
IL6 expression

## Slide 9
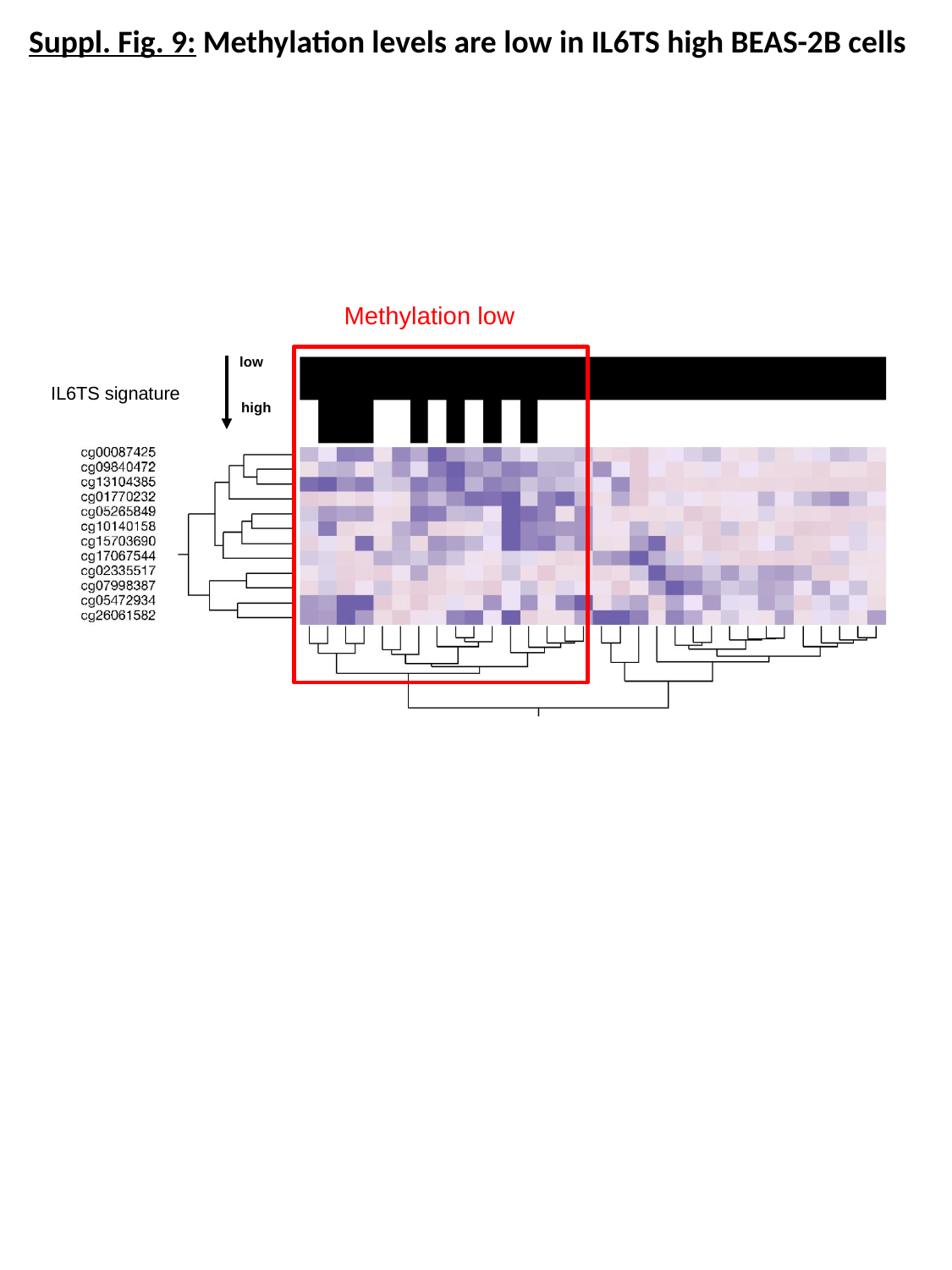

Suppl. Fig. 9: Methylation levels are low in IL6TS high BEAS-2B cells
Methylation low
low
high
IL6TS signature

## Slide 10
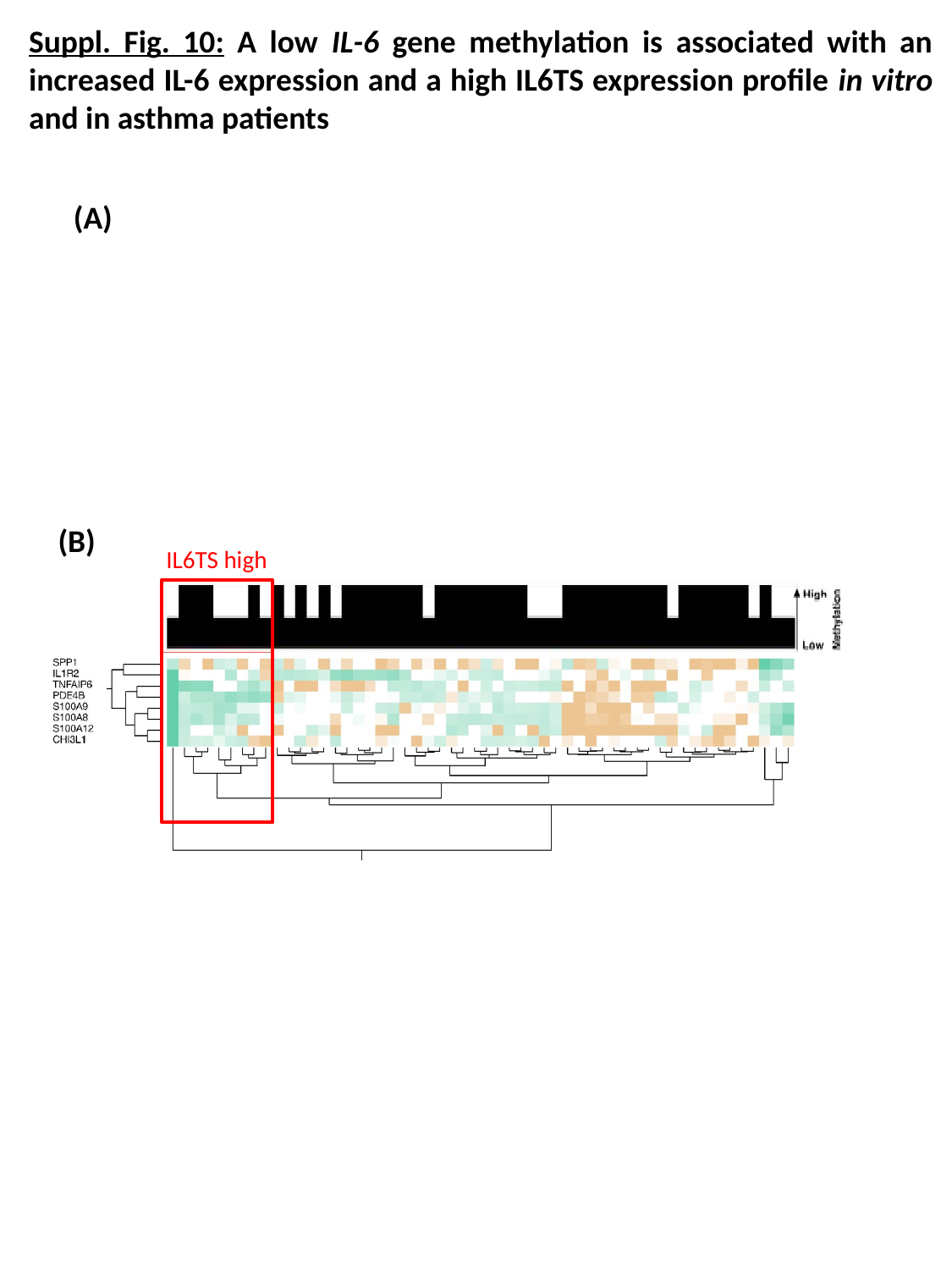

Suppl. Fig. 10: A low IL-6 gene methylation is associated with an increased IL-6 expression and a high IL6TS expression profile in vitro and in asthma patients
(A)
(B)
IL6TS high

## Slide 11
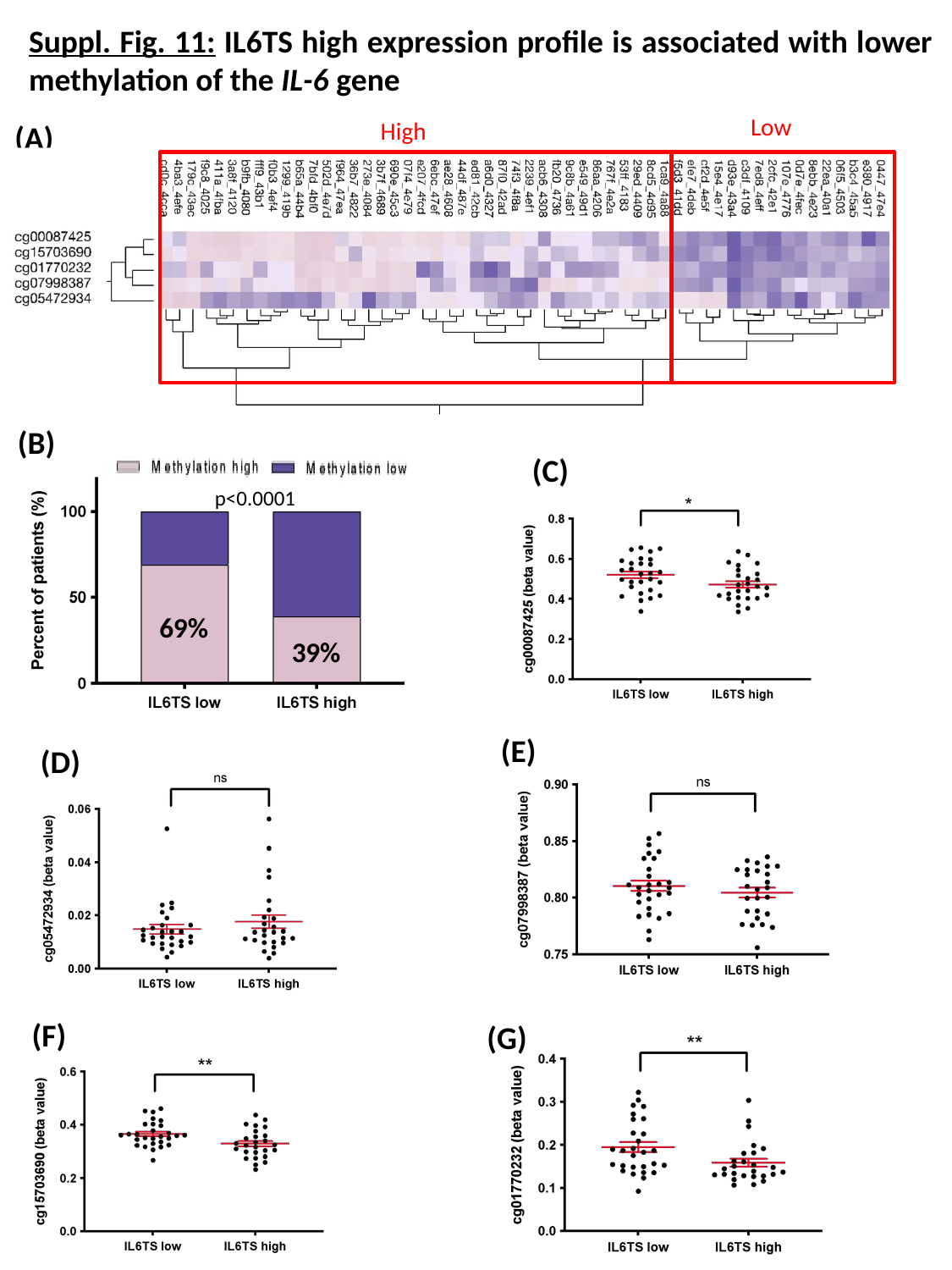

Suppl. Fig. 11: IL6TS high expression profile is associated with lower methylation of the IL-6 gene
Low
High
(A)
(B)
69%
39%
p<0.0001
(C)
(E)
(D)
(F)
(G)

## Slide 12
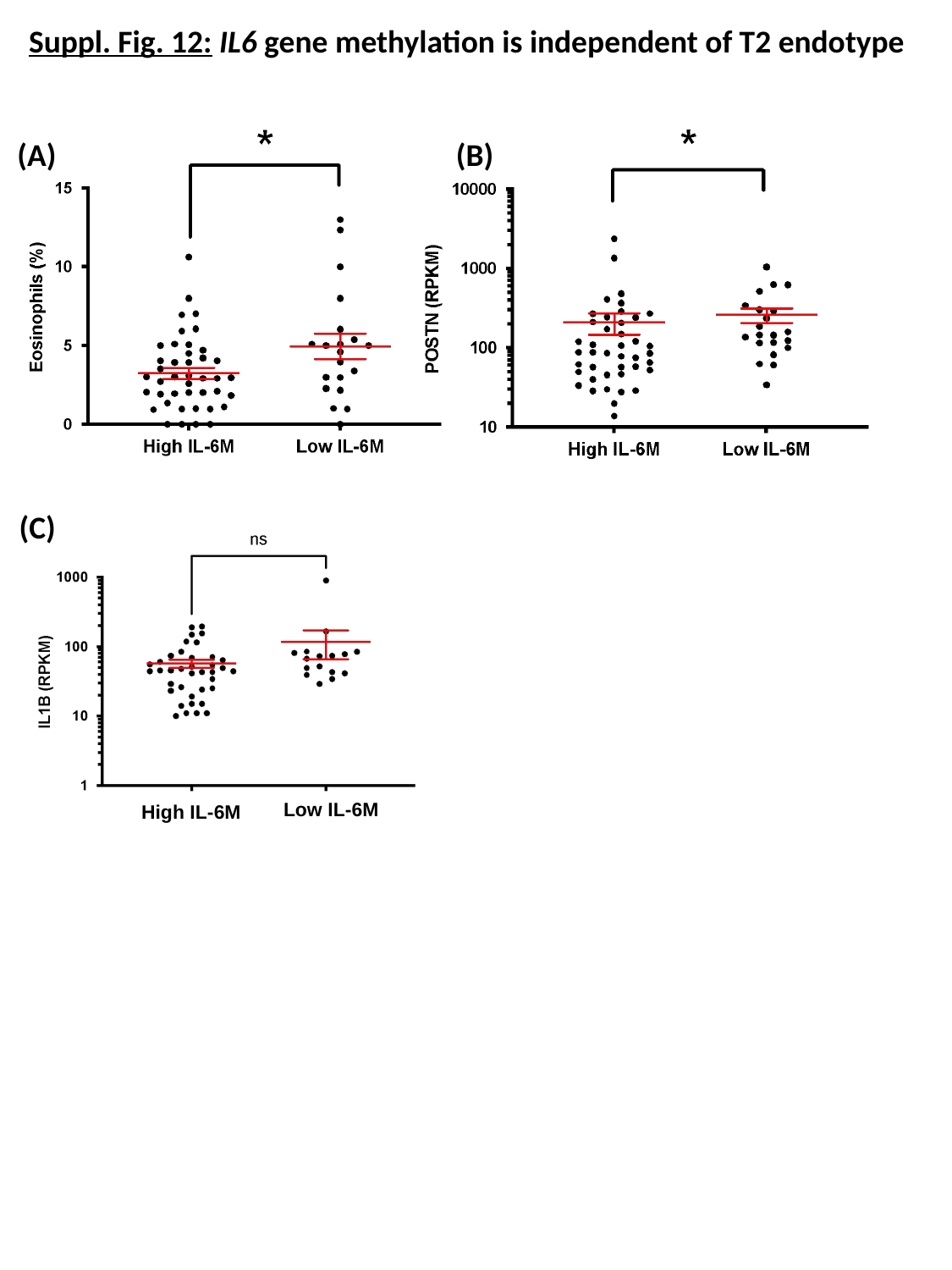

Suppl. Fig. 12: IL6 gene methylation is independent of T2 endotype
(A)
(B)
Low IL-6M
High IL-6M
(C)

## Slide 13
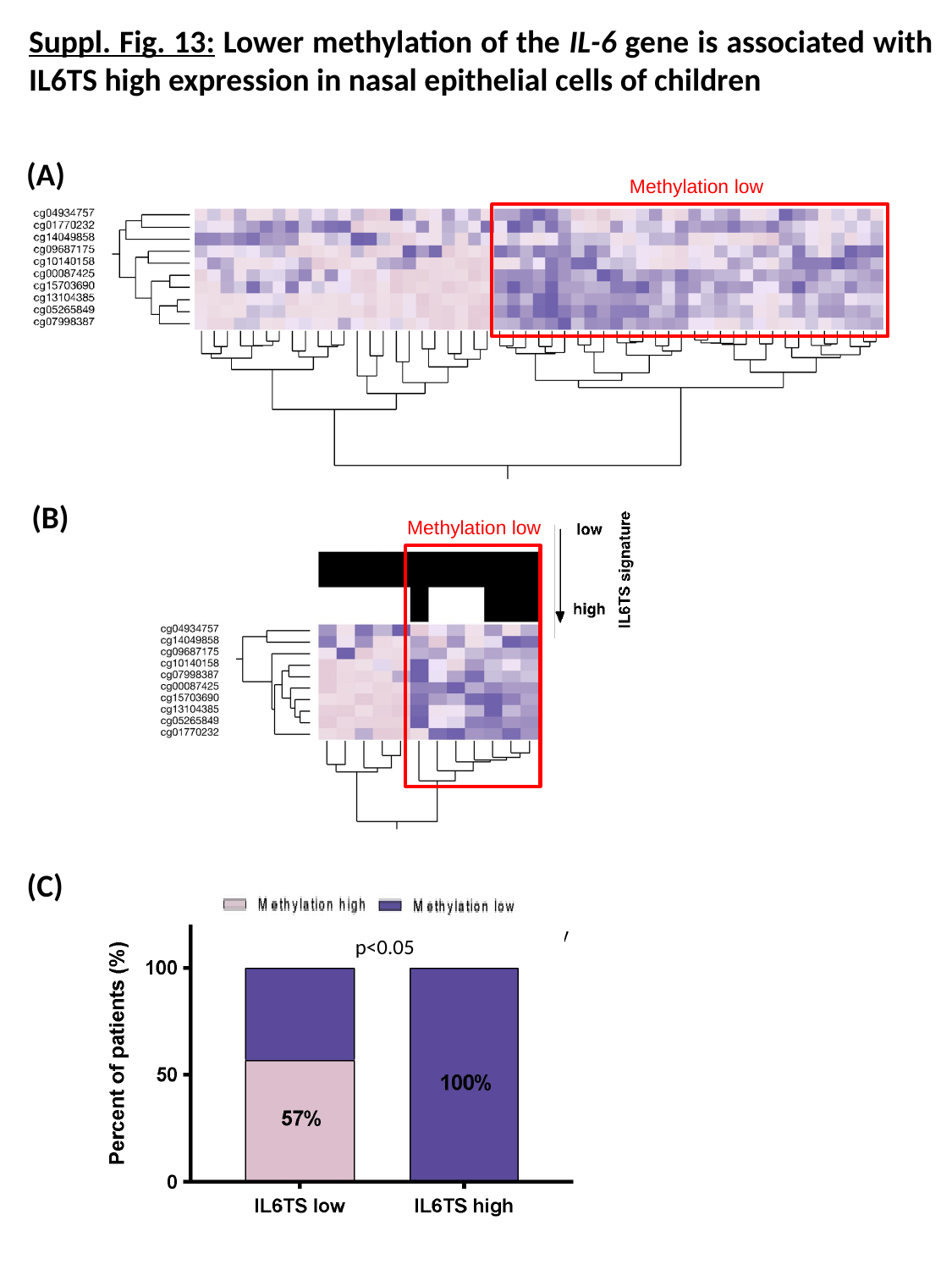

Suppl. Fig. 13: Lower methylation of the IL-6 gene is associated with IL6TS high expression in nasal epithelial cells of children
(A)
Methylation low
(B)
Methylation low
(C)
p<0.05

## Slide 14
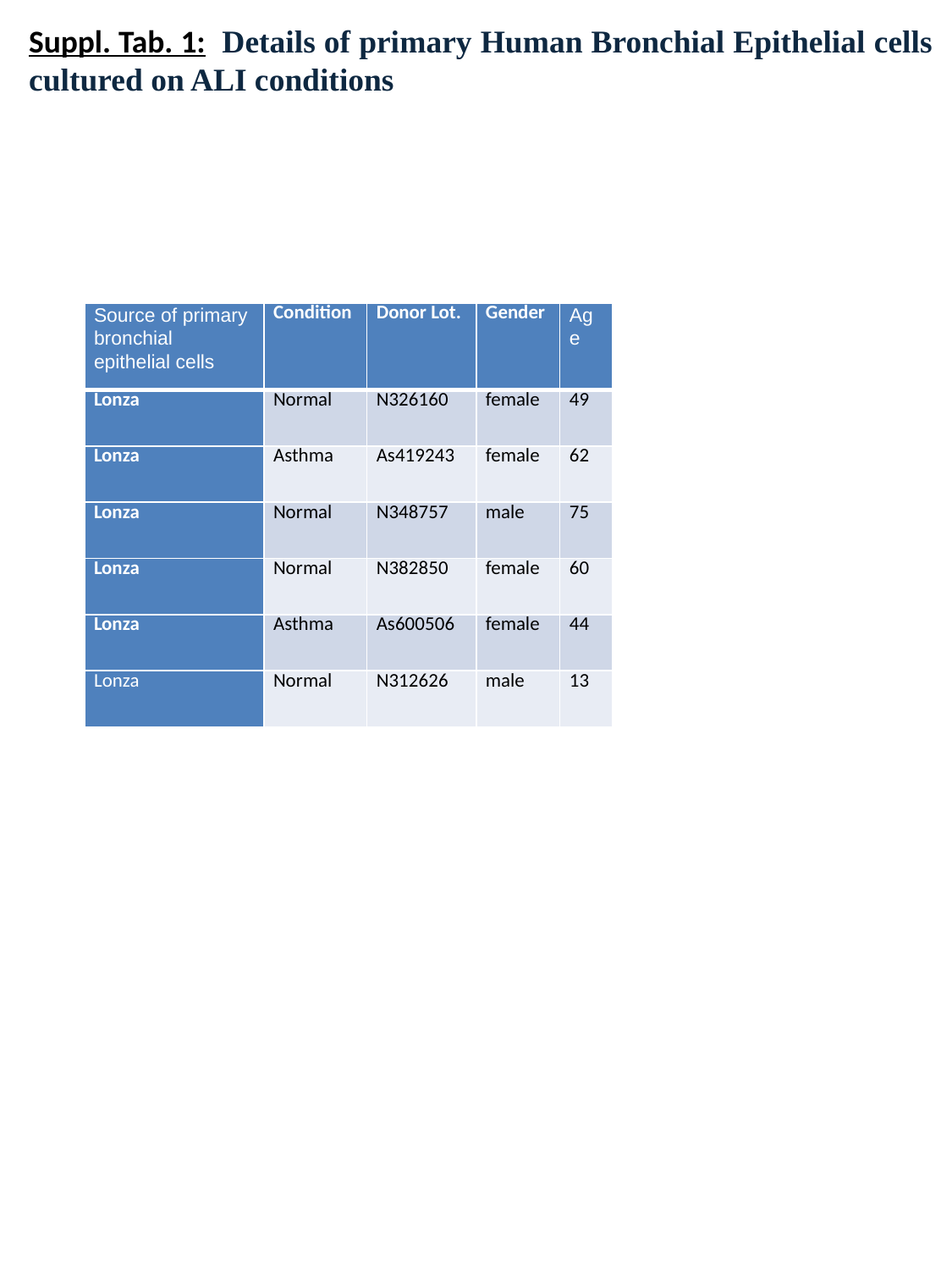

Suppl. Tab. 1: Details of primary Human Bronchial Epithelial cells cultured on ALI conditions
| Source of primary bronchial epithelial cells | Condition | Donor Lot. | Gender | Age |
| --- | --- | --- | --- | --- |
| Lonza | Normal | N326160 | female | 49 |
| Lonza | Asthma | As419243 | female | 62 |
| Lonza | Normal | N348757 | male | 75 |
| Lonza | Normal | N382850 | female | 60 |
| Lonza | Asthma | As600506 | female | 44 |
| Lonza | Normal | N312626 | male | 13 |

## Slide 15
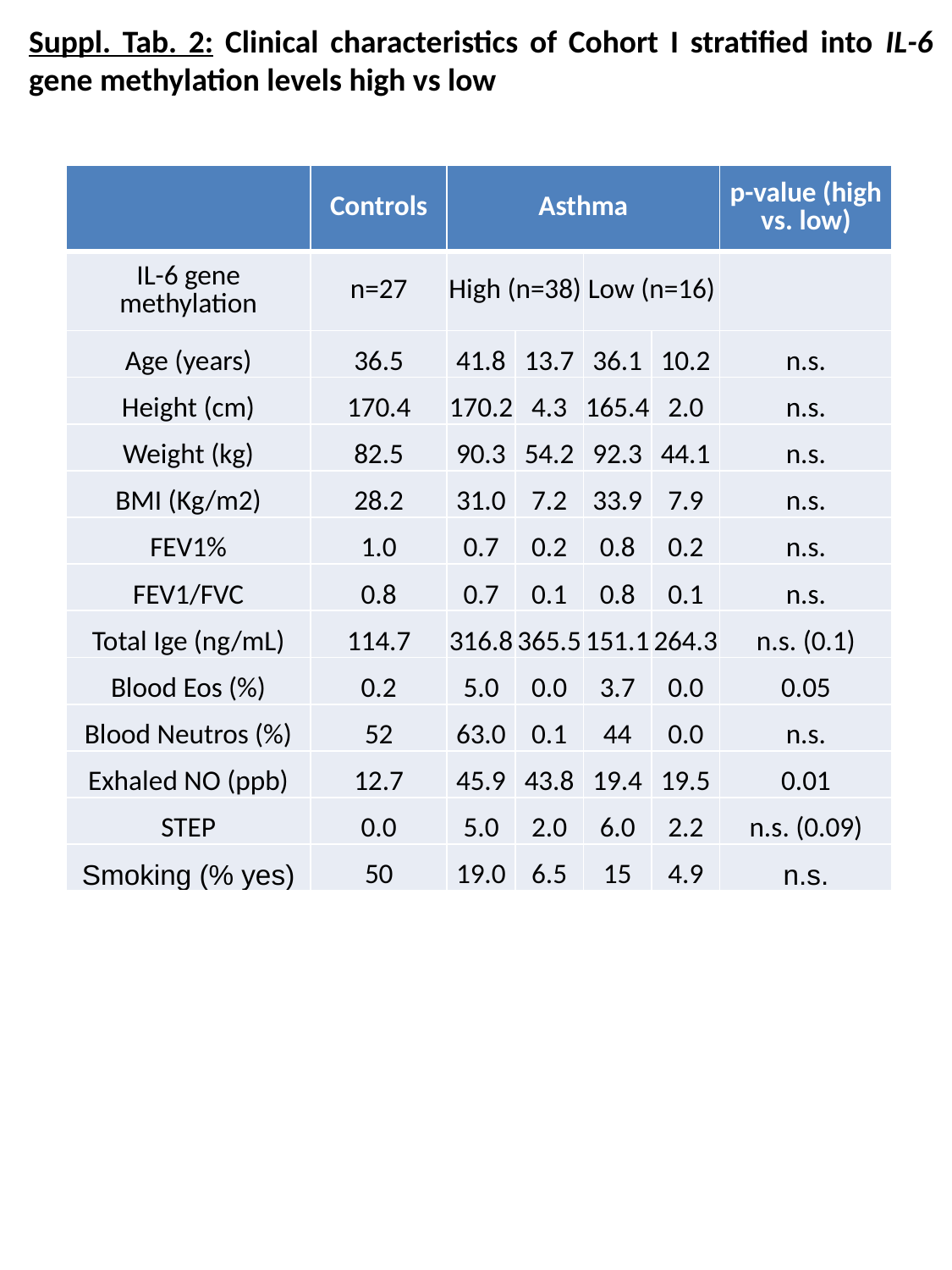

Suppl. Tab. 2: Clinical characteristics of Cohort I stratified into IL-6 gene methylation levels high vs low
| | Controls | Asthma | | | | p-value (high vs. low) |
| --- | --- | --- | --- | --- | --- | --- |
| IL-6 gene methylation | n=27 | High (n=38) | | Low (n=16) | | |
| Age (years) | 36.5 | 41.8 | 13.7 | 36.1 | 10.2 | n.s. |
| Height (cm) | 170.4 | 170.2 | 4.3 | 165.4 | 2.0 | n.s. |
| Weight (kg) | 82.5 | 90.3 | 54.2 | 92.3 | 44.1 | n.s. |
| BMI (Kg/m2) | 28.2 | 31.0 | 7.2 | 33.9 | 7.9 | n.s. |
| FEV1% | 1.0 | 0.7 | 0.2 | 0.8 | 0.2 | n.s. |
| FEV1/FVC | 0.8 | 0.7 | 0.1 | 0.8 | 0.1 | n.s. |
| Total Ige (ng/mL) | 114.7 | 316.8 | 365.5 | 151.1 | 264.3 | n.s. (0.1) |
| Blood Eos (%) | 0.2 | 5.0 | 0.0 | 3.7 | 0.0 | 0.05 |
| Blood Neutros (%) | 52 | 63.0 | 0.1 | 44 | 0.0 | n.s. |
| Exhaled NO (ppb) | 12.7 | 45.9 | 43.8 | 19.4 | 19.5 | 0.01 |
| STEP | 0.0 | 5.0 | 2.0 | 6.0 | 2.2 | n.s. (0.09) |
| Smoking (% yes) | 50 | 19.0 | 6.5 | 15 | 4.9 | n.s. |

## Slide 16
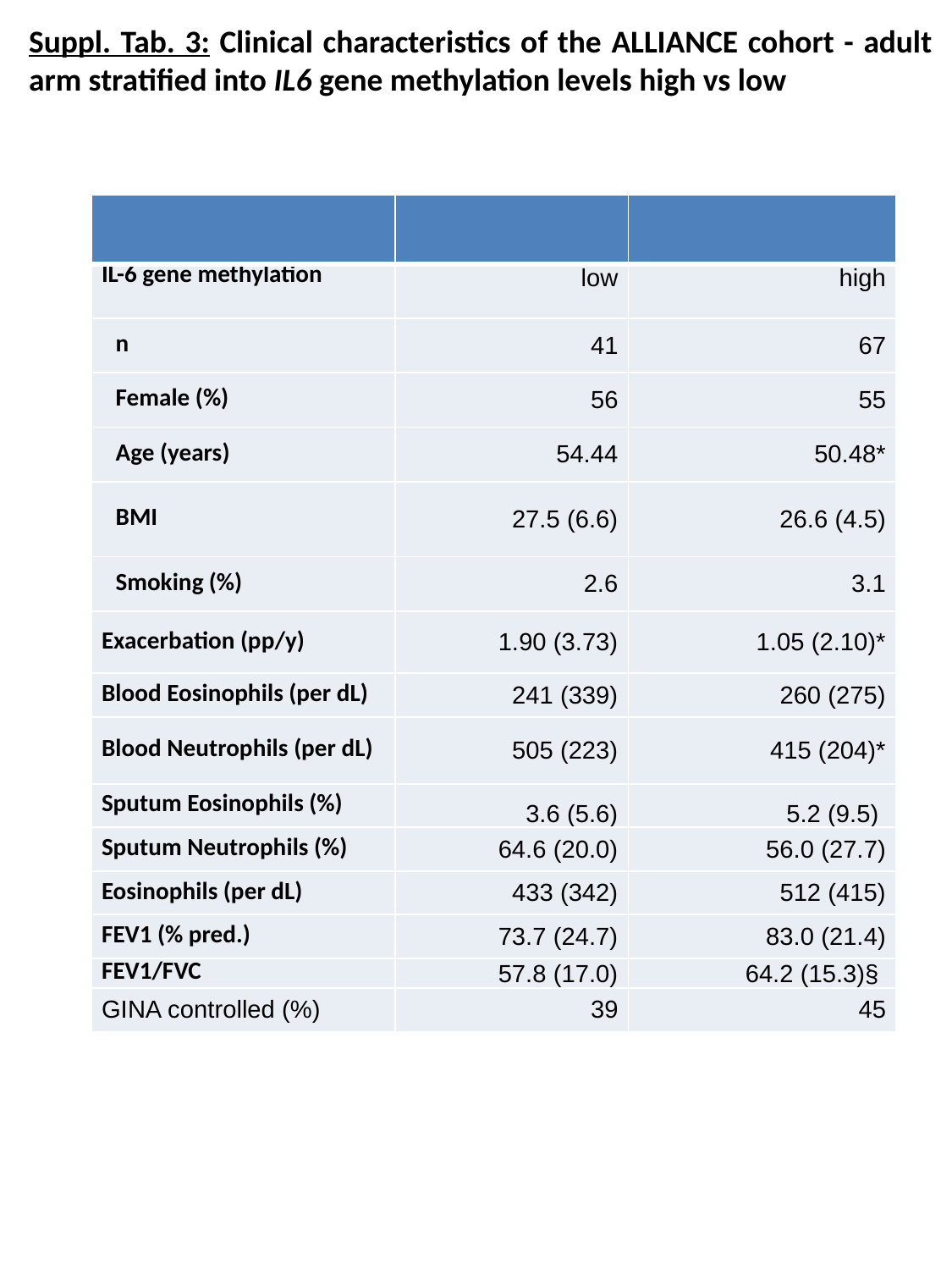

Suppl. Tab. 3: Clinical characteristics of the ALLIANCE cohort - adult arm stratified into IL6 gene methylation levels high vs low
| | | |
| --- | --- | --- |
| IL-6 gene methylation | low | high |
| n | 41 | 67 |
| Female (%) | 56 | 55 |
| Age (years) | 54.44 | 50.48\* |
| BMI | 27.5 (6.6) | 26.6 (4.5) |
| Smoking (%) | 2.6 | 3.1 |
| Exacerbation (pp/y) | 1.90 (3.73) | 1.05 (2.10)\* |
| Blood Eosinophils (per dL) | 241 (339) | 260 (275) |
| Blood Neutrophils (per dL) | 505 (223) | 415 (204)\* |
| Sputum Eosinophils (%) | 3.6 (5.6) | 5.2 (9.5) |
| Sputum Neutrophils (%) | 64.6 (20.0) | 56.0 (27.7) |
| Eosinophils (per dL) | 433 (342) | 512 (415) |
| FEV1 (% pred.) | 73.7 (24.7) | 83.0 (21.4) |
| FEV1/FVC | 57.8 (17.0) | 64.2 (15.3)§ |
| GINA controlled (%) | 39 | 45 |

## Slide 17
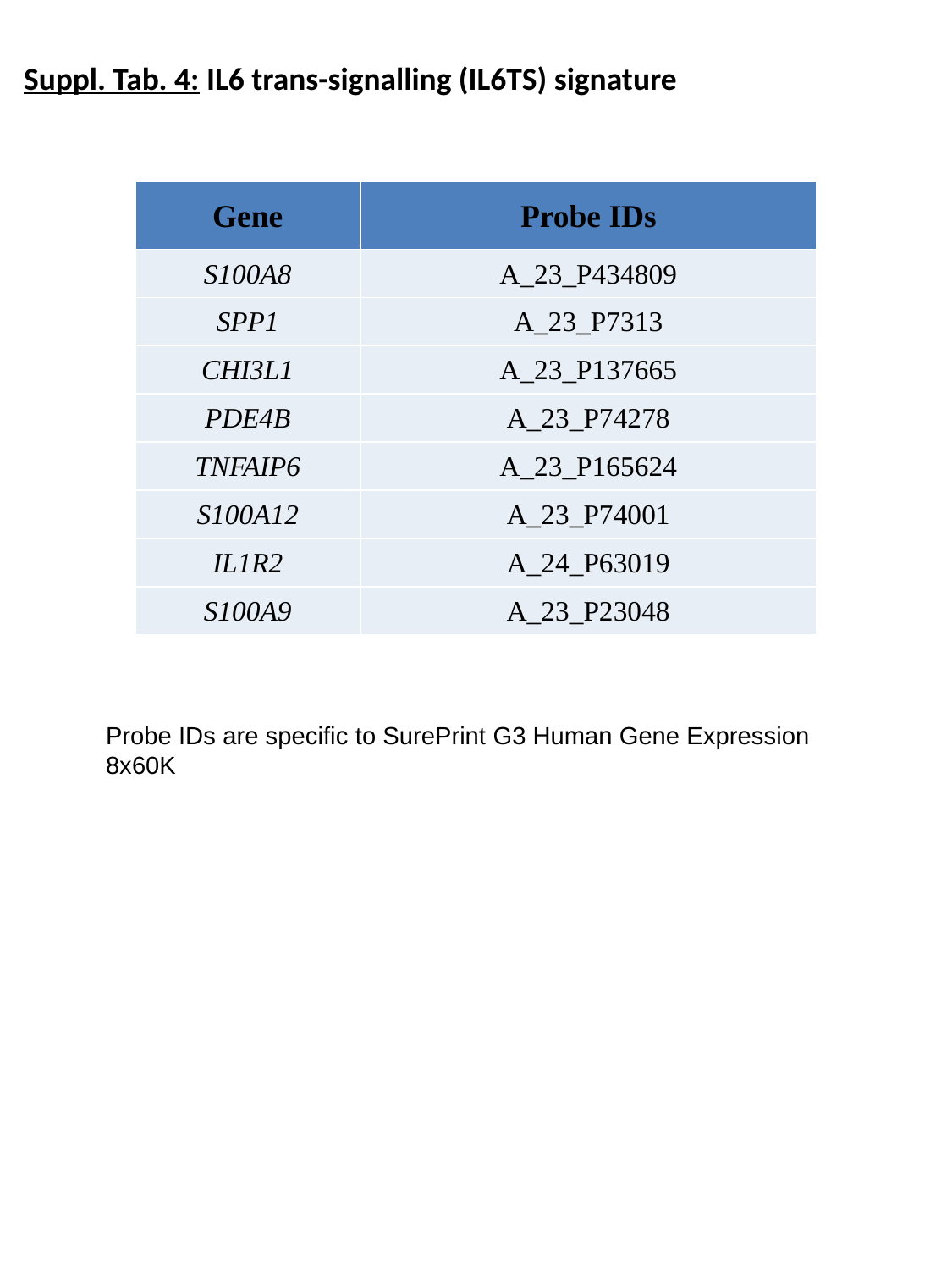

Suppl. Tab. 4: IL6 trans-signalling (IL6TS) signature
| Gene | Probe IDs |
| --- | --- |
| S100A8 | A\_23\_P434809 |
| SPP1 | A\_23\_P7313 |
| CHI3L1 | A\_23\_P137665 |
| PDE4B | A\_23\_P74278 |
| TNFAIP6 | A\_23\_P165624 |
| S100A12 | A\_23\_P74001 |
| IL1R2 | A\_24\_P63019 |
| S100A9 | A\_23\_P23048 |
Probe IDs are specific to SurePrint G3 Human Gene Expression 8x60K
