# Supplementary material for: Immune Training of the Interleukin 6 Gene in Airway Epithelial Cells is Central to Asthma Exacerbations
Source: Allergy. 2025 Oct 16;81(1):157–69. doi: 10.1111/all.70070 (PMC12773691; doi:10.1111/all.70070)
Supplement: Supplementary file 1 — Data S1: all70070‐sup‐0001‐Supinfo.zip. [file ALL-81-157-s001.zip › all70070-sup-0003-Supinfo1@Lunding_et_al._IL-6_suppl._file_revised_and_clean_20250721.docx]

**Immune training of the Interleukin 6 gene in airway epithelial cells is central to asthma exacerbations**

^Lars P. Lunding#, PhD, Prof. Markus Weckmann#, PhD, Ulrich M. Zissler, PhD, Constanze Jakwerth, PhD, Rebecca Bodenstein-Sgró, PhD, Sina Webering, PhD, Christina Vock, PhD, Johanna C. Ehlers, PhD, Romina A. M. Fernandez Ceballos, MSc, Sai Sneha P. Nemani MSc, Karosham Diren Reddy, PhD, Prof. Brian George G. Oliver, PhD, Cornelis J. Vermeulen, PhD, Maarten van de Berge, PhD, Prof. Carole Ober, PhD, Axel Künstner, PhD, Prof. Hauke Busch, PhD, Prof. Inke König, PhD, Prof. Christoph Garbers, PhD, Prof. Carsten B. Schmidt-Weber, PhD, Prof. Marcel F. Nold, MD, Ali Önder Yildirim, VD, Prof. Claudia A. Nold-Petry, PhD, Zane Orinska, PhD, Prof. Thomas Bahmer , MD, Prof. Jan Heyckendorf, MD, Prof. Gesine Hansen, MD, Prof. Erika von Mutius, MD, Prof. Klaus F Rabe, MD, Prof. Anna-Maria Dittrich, MD, Prof. Bianca Schaub,^ ^MD,^ ^Prof. Folke Brinkmann,^ ^MD, Prof. Matthias V. Kopp, MD, Michael Wegmann*, PhD and the ALLIANCE Study Group as part of the German Centre for Lung Research (DZL)^

^#Contributed equally^

*Corresponding Author:

Michael Wegmann, PhD

Division of Lung Immunology

Priority Area Chronic Lung Diseases

Research Center Borstel

Parkallee 1-40

23845 Borstel

Germany

Email: mwegmann@fz-borstel.de

Phone: (+49) 04537 - 188 5830

# Material & Methods

## *In-vivo* studies

## Animal treatment protocols

Female wild-type C57BL/6, interleukin (IL) 6 deficient (IL-6^-/-^)^1^, and IL-6 reporter (Il6^tm3307(Cerulean-P2A-CD90.1)Arte^) mice, aged 6–8 weeks, were housed under specific pathogen-free conditions receiving ovalbumin (OVA)-free diet and water *ad libitum*.

The sensitisation was induced by three intraperitoneal (i.p.) injections of 10 µg ovalbumin (OVA Grade VI, A2512, Sigma Aldrich, St. Louis, USA) dissolved in 100 µl PBS in combination with 100 µl of the adjuvant aluminium hydroxide (Al(OH)3) (Imject Alum, 77161, Thermo Fisher Scientific, Waltham, USA) on days 1, 14 and 21 of the experimental protocol. To induce a local acute allergic inflammatory response in the lung, on days 26-28 the animals were placed in an airtight chamber and exposed to an OVA aerosol (1% OVA grade V, A5503, dissolved in PBS, Sigma Aldrich) for 20 min. The OVA solution was inhaled by nebulisation using a generator (PARI® Master, Pari, Starnberg, Germany). To study the effects of TLR3 activation, as a subset of viral infection, on an already established allergic inflammatory response in the lung, 200 µg of polyinosinic-polycytidylic acid (poly(I:C), P9582, Sigma Aldrich) was dissolved in PBS and applied intranasally (i.n.) to the mice one hour after the last exposure to OVA on day 28. Animals were analysed 24 hours later on day 29. For IL-6 neutralisation, 70 µg anti–IL-6 monoclonal antibody (clone 20F3, MP5-20F3 InVivo BioTech, Hennigsdorf, Germany) or immunoglobulin (Ig) G1κ isotype control antibody (clone eBRG1, 14-4301-82 Invitrogen, Carlsbad, USA)^2^ dissolved in 70 µL sterile 0.9% NaCl solution was delivered via oropharyngeal application to mice on day 28. For repeated poly(I:C) exposures animals underwent the same protocol as for the acute exacerbation but were additionally exposed to an OVA aerosol on days 33, 34, 35, 40, 41, 42, 47 48, 49, 54, 55, and 56 and received four additional poly(I:C) applications on days 35, 42, 49, and 57. For poly(I:C) dosage experiments sevoflurane anaesthetized mice received 0, 2, 20, or 200 μg poly(I:C) by oropharyngeal application (o.a.) dissolved in 50 μL sterile phosphate-buffered saline (PBS) on day 28. All animals were sacrificed by cervical dislocation under deep anaesthesia. Sampling (serum, broncho-alveolar lavage (BAL), lung tissue) for kinetic experiments was performed at 2h, 4h, 8h, and 12 h, with all other acute exacerbation experiments sampled 24 h after the last poly(I:C) application. Animals for the negative control group were sham sensitised to PBS and subsequently challenged with ovalbumin (OVA) aerosol and were treated with PBS. Eight animals per group were used, unless stated otherwise.

**Bronchoalveolar lavage and differential cell count**

Lungs were lavaged with 1 mL ice-cold PBS containing protease inhibitor (Complete, 11697498001, Roche, Basel, Switzerland) via a tracheal cannula. Cells were counted using a Neubauer counting chamber. Aliquots of 50 µL of lavage fluids were cytospinned (Cytospin^TM^; Thermo Fischer Scientific), stained with Diff-Quick (720555-000, RAL Diagnostic, Martillac, France), and cells were microscopically differentiated according to morphologic criteria as previously described^3^.

**Assessment of airway hyperresponsiveness (AHR)**

Airway responsiveness to methacholine (acetyl-β-methylcholine chloride, A2251, Sigma-Aldrich, St. Louis, MO, USA) challenge was invasively assessed in anesthetised and ventilated mice on day 29 using FinePointe RC Units (Data Science International, St. Paul, MN, USA) by continuous measurement of airway resistance (RI). Animals were anesthetised with ketamine (90 mg/kg body weight; 1202, cp-pharma, Burgdorf, Germany) and xylazine (10 mg/kg BW; 1205, cp-pharma, Burgdorf, Germany) and tracheotomised with a cannula, with mechanical ventilation as previously described^3^. Measurements were taken at baseline (PBS) and in response to inhalation of increased concentrations of aerosolised methacholine (3.125; 6.25; 12.5; 25; 50; and 100 mg/mL). After assessment of lung function, all animals were sacrificed by cervical dislocation under deep anaesthesia.

**Lung histology**

Mouse lungs were inflated and fixed *ex-situ* by instillation of 4% (w/v) phosphate-buffered paraformaldehyde (P087.1, Carl Roth, Karlsruhe, Germany) under constant pressure for 20 min. Subsequently, the lung was kept at 4°C in paraformaldehyde overnight and was embedded in paraffin. The orientation of the lungs was randomised according to the orientator technique^4^. For analysis of lung inflammation, 2 μm sections were stained with periodic acid-Schiff (PAS, 1.09033, Merck Millipore, Burlington, MA, USA). For histological staining of IL-6, 2 μm sections of IL-6 reporter mice lungs were immunohistochemically stained using an CD90.1 antibody (ab3105, Abcam, Cambridge, UK; 1:100) as primary antibody to detect the reporter gene. Prior to the staining against CD90.1, sections were heated in citrate buffer (pH 6) for 20 min. After pre-treatment, sections were incubated with the primary antibody for 1 h at 37°C. Bound antibodies were visualised using biotinylated secondary goat anti-rabbit antibody (BA-1000, Vector Laboratories, Newark, CA, USA) followed by avidin peroxidase (PK-6100, Vectastain Elite kit, Vector Laboratories, Newark, CA, USA) and 3,3′-diaminobenzidine (DAB) substrate (D1238-4, Sigma-Aldrich). Afterwards, samples were counterstained with Mayer's haematoxylin (1.092491000, Merck Millipore). For negative controls, buffer was used instead of the first antibody. Photomicrographs were recorded by a digital camera (DP-25; Olympus, Tokyo, Japan) attached to a microscope (BX-51, Olympus, Tokyo, Japan) with a 20-fold magnification objective using Olympus cell A software. Quantification of staining was scored using the Quick score (Q-score), which is calculated by multiplying the intensity score (0-3) and the proportion score (0-4). Quantification was done by two different blinded investigators.

**Quantitative morphology**

PAS was used to stain mucus in the airways. For mucus assessment, systematic uniform random samples of lung tissue were taken according to standard methods^5^. The surface area of mucin-containing goblet cells (S_gc_) per total surface area of airway epithelial basal membrane (S_ep_) and the volume of PAS-stained stored epithelial mucus (V_mucus_) per S_ep_ were determined using a computer-assisted stereology tool box (newCAST, Visiopharm, Hoersholm, Denmark)^6,7^ according to the following formulas: $S_{gc}/S_{ep}=\Sigma I_{gc}/\Sigma I_{ep}$ and $\frac{V_{mucus}}{S_{ep}=LP\times\frac{\Sigma P_{mucus}}{2\times\Sigma I_{ep}}}$ where $\Sigma I_{gc}$ is the sum of intersections of test lines with goblet cells; $\Sigma I_{ep}$ is the sum of all intersections of test lines with epithelial basal membrane; $\Sigma P_{mucus}$ is the sum of all points hitting mucus and LP is the test line length at final magnification^8^.

**FACS analysis of leukocytes from the lung**

The dissected rinsed lung was cut into pieces and incubated in 10ml digestion mix for 30min at 37°C and agitation (180rpm). The digestion mix containing 30µg/ml DNAse I from bovine pancreas and 0.7mg/ml collagenase from Clostridium histolyticum in RPMI 1640 cell culture medium supplemented with 10% FCS, 10mM HEPES, 1mM sodium pyruvate, 10U/ml penicillin, 0.1mg/ml streptomycin, 50mM 2b-mercapthoethanol was added to the minced lung tissue. After digestion, cell suspension was grated through a 100µm mesh and washed with 15ml 3%FCS in HBSS. Cells were centrifuged for 5min at 472xg and 6°C. Cell pellet was resuspended in ACK-Lyse buffer (0.15M NH4CL, 10mM KHCO3, 100µM EDTA, pH7.2) to lyse erythrocytes for 2min at RT. Cells were washed with 30ml 3%FCS in HBSS. Cell pellet was resuspended in 3ml 3% FCS in HBSS, overlayed on 3ml Lympholyte cell separation medium and centrifuged 1200xg for 20min at RT without brake. Cells on the interphase were collected, washed with 3% FCS in HBSS, resuspended in FACS buffer, filtered through a 30µm mesh and cell concentration determined.

1 x 10^6^ cells pro sample were preincubated with FcBlock in FACS buffer in 96 well plate for 10min on ice. After incubation, staining mix containing fluorescently labeled antibodies (see table) was added and cells were incubated for 30 min on ice. To detect NKT cells, BV421 labeled CD1d tetramers complexed with PBS-57 were used. Cells were washed with FACS buffer by centrifugation at 800xg for 3min, before staining with fluorescent streptavidine (Brilliant Violet 605™ Streptavidin, BioLegend) or washed with PBS and stained with fixable viability dye (LIVE/DEAD™ Fixable Blue Dead Cell Stain, Invitrogen) for 15min at RT. Cells were washed with FACS buffer and fixed in 2% PFA solution for 10min at RT, afterwards washed in and resuspended in FACS buffer until measurement. Samples were measured at LSR II (BD Biosciences) and data analyzed with FlowLogic (Inivai).

| **Antigen** | **Clone** | **Fluorochromes** | **Catalog Numbers** | **Dilution** | **Vendor** |
| --- | --- | --- | --- | --- | --- |
| CD45 | 30-F11 | APC-Cy7 | 103116 | 1:500 | BioLegend |
| CD11b | M1/70 | PE-Cy7 | 101216 | 1:500 | BioLegend |
| CD19 | 1D3 | BUV737 | 564296 | 1:250 | BD Biosciences |
| TCRβ | H57-597 | FITC | 109206 | 1:300 | BioLegend |
| NK1.1 | PK136 | PE  BV421 | 108708,  108741 | 1:300  1:300 | BioLegend  BioLegend |
| CD8α | 53-6.7 | BV785 | 100750 | 1:300 | BioLegend |
| CD4 | RM4-5 | PerCP Cy5.5 | 100504 | 1:500 | BioLegend |
| TCRγδ | GL3 | BV605 | 118129 | 1:300 | BioLegend |
| CD127 | A7R 34 | Vio®Bright FITC | 130-116-502 | 1:50 | Miltenyi Biotec |
| CD117 | 2B8 | BV711 | 105835 | 1:500 | BioLegend |
| CD90.2 | 30-H12 | Biotin | 105304 | 1:500 | BioLegend |
| CD11c | N418 | APC | 117310 | 1:300 | BioLegend |
| CD45R/B220 | RA3-6B2 | BV510 | 103248 | 1:500 | BioLegend |
| Siglec F | E50-2440 | PE | 552126 | 1:300 | BD Biosciences |
| CD62L | MEL-14 | BV711 | 104445 | 1:500 | BioLegend |
| CD3ε | 145-2C11 | BUV737 | 564618 | 1:300 | BD Biosciences |
| FcBlock CD16/32 | 93 | unlabeled | 101302 | 1:50 | BioLegend |

**Assessment of cytokines**

Levels of murine IL-1β, IL-4, IL-5, IL-6, IL-13, and IL-17A in bronchoalveolar lavage (BAL) or serum samples were assessed using enhanced cytometric bead array (Flex Set Kits; BD Bioscience, Franklin Lakes, NJ, USA) and BD Accuri^TM^ C6 flow cytometer (BD Bioscience, Franklin Lakes, NJ, USA) according to the manufacturer´s guidelines. The analysis was performed using FCAP Array^TM^ v3.0 Software (BD Bioscience, Franklin Lakes, NJ, USA). Levels of murine eotaxin-1, IFN-α, IFN-β, IFN-λ3, KC, and VEGF in BAL fluid were determined using enzyme-linked immunosorbent assay (ELISA) kits (Eotaxin Mouse ELISA Kit, Abcam, Cambridge, UK; IFN-α Mouse ELISA, Affymetrix, Thermo Fisher Scientific, Waltham, USA; IFN-β Mouse ELISA, BioLegend, San Diego, CA, USA; IFN-λ3 Mouse ELISA, Invitrogen, Thermo Fisher Scientific, Waltham, MA, USA; KC Mouse ELISA, R&D Systems, Minneapolis, MN, USA; VEGF Mouse ELISA , R&D Systems, Minneapolis, MN, USA) according to manufacturer’s guidelines. The analysis was performed by determination of absorption at 450 nm using a photometer (Sunrise, Tecan, Männedorf, Switzerland) and Magelan 7 Software (Stüber Systems, Berlin, Germany).

Levels of human IL-6 were measured in tissue culture supernatant as previously reported^9^. In brief, a bead ELISA Bio-Plex Pro™ Human Chemokine 40-plex Assay was used in conjunction with a Bio-Plex MAGPIX Multiplex Reader (both BioRad, Hercules, CA, USA), as per manufacturer’s specifications.

**ELISA of soluble murine IL-6R**

For the enzyme-linked immunosorbent assay (ELISA), soluble murine IL-6 receptor was quantified using Mouse IL-6R alpha DuoSet ELISA (R&D Systems, Minneapolis, MN, USA) according to the manufacturer's instructions. Serum samples were diluted prior to ELISA measurement according to the assay range of the ELISA.

**Reverse transcription and quantitative real-time PCR for murine samples**

Ribonucleic acid (RNA) was isolated using RNeasy® Micro Kit (74104, Qiagen, Hilden, Germany) and reversely transcribed into copy deoxyribonucleic acid (cDNA) using Maxima First Strand cDNA Synthesis Kit (K1671, Thermo Fisher Scientific) according to the manufacturer’s guidelines. Real-time Polymerise chain reaction (PCR) was performed on a LightCycler 480 II instrument (Roche Applied Science, Mannheim, Germany) according to the manufacturer’s instructions with the Light Cycler 480 Sybr Green II Master (04707516001, Roche Applied Science, Mannheim, Germany) in a total volume of 10 µL. Cycling conditions were as follows: one cycle at 95°C for 10 min followed by 45 cycles touch-down 63°C–58°C for 8 s with 0.5°C/sec and 72°C for 10 sec. For each primer pair, a standard curve was established by serial cDNA dilutions. RPL-32 was used as housekeeping gene.

Following primers were used: *IL-6* forward 5′- CTCCCAACAGACCTGTCTATAC-3′, reverse 5′- GTGCATCATCGTTGTTCATAC-3′. Data were analysed employing the ‘advanced relative quantification’ and ‘standard curve method’. A calibrator cDNA was included in each run to correct for run-to-run differences. Amplification specificity was checked using melting curve. PCR products were visualized by agarose gel electrophoresis.

**Human rhinovirus (RV) 16 Propagation**

Human RV16 was kindly provided by Prof. Dr. Brian Oliver (Woolcock Institute for Medical Research, Sydney, Australia). Human RV-16 was propagated in HeLa cells (ATCC, Manassas, VA, USA), centrifuged with a 10% Sarcosyl (N-Lauroylsarcosine sodium salt solution, Sigma-Aldrich, Deisenhof, Germany), and then separated with a sucrose gradient (60%, 45%, 30%, 15%, Sucrose, Sigma-Aldrich, Deisenhof, Germany). Final virus stock was tested for multiplicity of infection (MOI) by a plaque assay.

**Viral transcript analysis**

RV16 transcripts in RNA-Seq data were detected by mapping each paired-end transcriptome to viral CDS (NCBI Accession: NC_001490.1 generated using *"R*" software (v4.3.1) with the package *bioconductor* (v3.18) and *subread* (v2.16.1). Total detected fragments and mapped fragments were calculated for each sample.

**Repeated RV16 infection and poly(I:C) exposure**

BEAS2-B cells (CRL-9609, ATCC, Manassas, VA, USA) were seeded at 2x10^6^ per T25 (Sarstedt, Nümbrecht, Germany) flask in bronchial epithelial cell growth medium (BEGM) without Gentamicin sulphate-Amphotericin (GA-1000) (Lonza, Visp, Switzerland) and given time to reach 80% confluence (24h). Cells were then infected with MOI 0 (vehicle) and 5^10^, or incubated with 5 µg poly(I:C) for 24 hours. After infection/exposure, cells were washed three times with Fetal bovine serum (FBS)-PBS and twice with PBS before enzymatic removal (Trypsin EDTA, PAA Laboratories, Coelbe, Germany) and centrifugation. Obtained cells were split, with 80% used for analysis (RLT-Buffer, Qiagen, Hilden, Germany) and 20% re-seeded virus- and poly(I:C)-free (PCR supernatant) for following infections/exposures. This procedure was repeated up to three (poly(I:C)) or five times with samples analysed after infections one, three and five or exposure one and three, respectively.

**Air-liquid interface (ALI) culture of human bronchial epithelial (HBE) cells**

Normal (N)HBEs and diseased (D)HBEs primary cells from five donors with asthma were purchased from Lonza (CC-2540S, 00194911S respectively, Lonza, Walkersville, MD, USA). Cells were grown in BEGM media according to the manufacturer’s instructions. When 80% confluency was reached, cells were detached (ReagentPack Subculture Reagents, CC-5034, Lonza, Walkersville, MD, USA) and seeded in collagen-coated (Collagen IV from human placenta, C5533, Sigma-Aldrich) transwell filters (CLS3401-48EA, Corning, Kennebunk, ME, USA). Cells were cultured with BEGM until confluence was reached. To perform the airlift, the apical medium was removed, and the basal medium was switched to PneumaCult ALI Medium (05001, Stemcell, Vancouver, Canada) prepared with 100 U/mL penicillin and 0.1 mg/mL streptomycin (P06-07100, PAN Biotech, Aidenbach, Germany). ALI-cultures were treated with 5 ng/mL recombinant human (rh) IL-13 (200-13-1MG, Peprotech, Rocky Hill, NJ, USA) from day 21 until day 28. On day 28, ALI-cultures were stimulated with 5 µg/mL poly(I:C) (Sigma-Aldrich) for 3 h. All stimulations were performed from the basal site.

**RNA isolation from human cells and whole genome microarray**

RNA from nasal brushings was extracted using RNeasy Micro Kit (Qiagen, Hilden, Germany) with on-column DNase digestion (Qiagen, Hilden, Germany) to avoid DNA contamination. RNA quantification was performed by ultraviolet–visible spectrophotometry (Nanodrop Technologies, Wilmington, USA), and assessment of the RNA integrity was performed by the RNA 6000 Nano Chip Kit with the Agilent 2100 Bioanalyzer (Agilent Technologies, Waldbronn, Germany). Total RNA was amplified and Cy3-labeled by using the one-color Low Input Quick Amp Labeling Kit (Agilent Technologies, Waldbronn, Germany) as described before according to the manufacturer’s protocol^11^. Hybridisation to SurePrint G3 Human Gene Expression 8x60K Microarrays (Agilent Technologies, Waldbronn, Germany) was performed with the Gene Expression Hybridization Kit (Agilent Technologies, Waldbronn, Germany). Data were imported using a standard baseline transformation to the median of all values, including log transformation and computation of fold changes. Subsequently, a principal component analysis (PCA) was conducted, which revealed a homogeneous component distribution. Compromised array signals were excluded from further analysis (the array spot is considered non-uniform if the pixel noise of a feature exceeds the threshold or is above the saturation threshold). Manhattan cityblock on entities (Ward’s linkage) was used to cluster changes in gene expression. ‘Arbitrary units’: Gene expression normalisation of the is performed by the pre-processing software pipeline of the Affymetrix SurePrint G3 gene expression arrays. All values are transformed to the global median, including log2 transformation and computation of fold changes. Gene expression levels (arbitrary units) are therefore the positive or negative log2 fold change of the median of the respective gene as compared to the global median.

**DNA Methylation and RNA expression analysis**

DNA methylation analysis and RNA sequencing were carried out at the Institute of Clinical Molecular Biology, Christian-Albrechts-University, Kiel, Germany, following the manufacturer's protocols. Genome wide analysis of locus-specific DNA methylation was performed using 500 ng bisulphite-converted genomic DNA (EZ DNA methylation Kit, Zymo Research, Freiburg, Germany) and the HumanMethylation450 BeadChip Kit (Illumina, WG-314-1003, München, Germany). β-values were normalized using Illumina’s algorithms (Partek Genomic Suite, Partek, Chesterfield, MO, USA). For RNA sequencing, 1000 ng of total RNA were applied with the HiSeq PE Cluster Kit v4, cBot (Illumina, PE-401-4001, San Diego, CA, USA), the HiSeq SBS Kit v4 (Illumina, FC-401-4003, San Diego, CA, USA) and the HiSeq2500 System (Illumina, San Diego, CA, USA). Strand-specific sequencing with 2x125 bp resulted in FASTQ data files further processed with Partek Flow (Partek, Chesterfield, MO, USA). Average read quality (Phred) was set to be >20 to optimise alignment. Alignment was performed using GSNAP (v3, 31.03.2013). All data are shown as reads per kilobase per million mapped reads (RPKM).


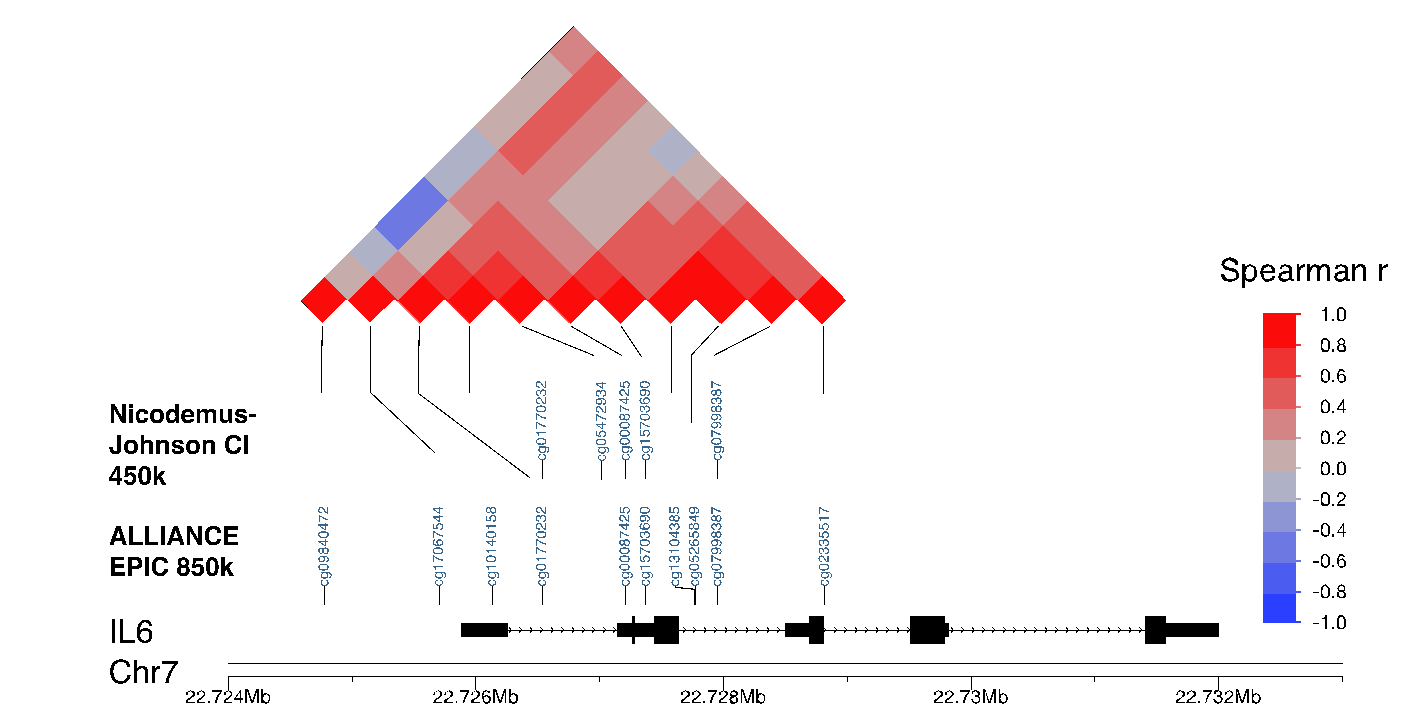


Genomic location map of DNA methylation of the IL6 gene. CpG correlation map (Spearman r) derived from <https://portal.gdc.cancer.gov> for Lung Adenocarcinoma (LUAD) and Squamous Cell Neoplasms (LUSC) with n=843 individual biosamples.

## *Ex-vivo* studies

## ALLIANCE cohort: paediatric and adult arm

The study was registered at ClinicalTrials.gov ([NCT02496468](https://clinicaltrials.gov/ct2/show/NCT02496468) for paediatric arm, [NCT02419274](https://clinicaltrials.gov/ct2/show/NCT02419274) for adult arm). The clinical dataset version is 20210730_V5-0.

# Supplementary figure legends

**Suppl. Fig. 1: Flow chart**

Flow chart of the paediatric & adult arm of ALLIANCE cohort. Final sample size was n=53 for the paediatric cohort and n =103 for the adult cohort.

**Suppl. Fig. 2: Kinetics of cytokine concentrations in BAL fluid upon exacerbation of experimental asthma in mice**

Concentration in BALF of PBS (n=8), PBS + poly I:C (n=8), OVA (n=8), or OVA + poly(I:C) mice (n=8) at five different time points (0, 2, 4, 8, and 12 h) after poly(I:C) exposure: Eotaxin-1 **(A)**, IFN-α **(B)**, IFN-β **(C)**, IFN-λ3 **(D)**, IFN-γ **(E)**, IL-1α **(F)**, IL-1β **(G)**, IL-4 **(H)**, IL-5 **(I)**, IL-6 **(K)**, IL-13 **(L)**, IL-17A **(M)**, KC **(N)**, TNF-α **(O)**, VEGF **(P)**. Results are presented as mean values ± S.E.M.. Statistical significance was assessed using ordinary one-way ANOVA and Tukey´s multiple comparison post-hoc analyses, *p<0.1, **p<0.01, ***p<0.001, and ****p<0.0001, n.s. = not significant. Only statistical differences of exacerbated vs asthmatic are depicted.

**Suppl. Fig. 3: Cytokine concentrations in BALF upon excaerbation of experimental asthma in WT, IL-6 deficient mice and anti-IL-6 treated mice**

Concentration in BALF of PBS, OVA, or OVA + poly(I:C) mice 24h after poly(I:C) exposure in wildtype or IL-6 deficient mice **(A-I)** or treated with or without an anti-IL-6 antibody **(J-R)**: IL-1α **(A, J)**, IL1-β **(B, K)**, IL-5 **(C, L)**, IFN-α **(D, M)**, IFN-γ **(E, N)**,  IFN-λ **(F, O)**, TNF-α **(G, P)**, VEGF **(H, Q)**, Eotaxin **(I, R)**. Results are presented as mean values ± S.E.M.. Statistical significance was assessed using ordinary one-way ANOVA and Tukey´s multiple comparison post-hoc analyses, *p<0.1, **p<0.01, ***p<0.001, and ****p<0.0001, n.s. = not significant. Only statistical differences of different exacerbated groups are depicted.

**Suppl. Fig. 4: poly(I:C) dose-dependency of allergic airway inflammation and IL-6 release in mice with experimental asthma**

**(A)** Numbers of macrophages, lymphocytes, neutrophils, and eosinophils in BALF of asthmatic mice (OVA, n=8), or asthmatic mice receiving low (OVA+poly(I:C) low, n=8), medium (OVA+poly(I:C) med, n=8), or high (OVA+poly(I:C) high, n=8) dose of poly(I:C) intra-nasally. **(B)** Concentration of IL-6 in BALF. Results are presented as mean values ± S.E.M.. Statistical significance was assessed using ordinary one-way ANOVA and Tukey´s multiple comparison post hoc analyses, *p<0.1, **p<0.01, ***p<0.001, and ****p<0.0001, n.s. = not significant.

**Suppl. Fig. 5: Immunohistochemical staining of IL-6 producers in the lung**

Immunohistochemical staining against IL-6 reporter gene product (CD90.1) of lung cross-sections. Scale bar, 50 µm. Representative staining of healthy (PBS) control mice and mice with stable (OVA) vs. exacerbated EAA (OVA + poly(I:C), and negative control (ab. control)). Stained alveolar type II cells (AT2), endothelial cells (EC), neutrophils (Neu), and macrophages (MΦ) are marked by black arrows. Histological Q-score of the staining in different areas of lung cross-sections (endothelium, epithelium, and inflammatory cell infiltrates). Results are presented as mean values ± S.E.M.. Statistical significance was assessed using ordinary one-way ANOVA and Tukey´s multiple comparison post hoc analyses, *p<0.1, **p<0.01, ***p<0.001, and ****p<0.0001, n.s. = not significant.

**Suppl. Fig. 6: FACS analysis of fast-responding innate immune cells infiltrating the lungs of mice with exacerbation of experimental asthma**

(A) Gating strategy for all viable CD54+ single cells. (B) Gating strategy for NKT cells, NK cells, and γδT cells. (C) Gating strategy for ILCs as indicated in purple. (D) Percentage of total leukocytes in the lung of γδT cells, NK cells, NKT cells, and ILCs. (E) Total number of γδT cells, NK cells, NKT cells, and ILCs from the lungs of PBS (n=6), OVA (n=6), and OVA + poly(I:C) mice (n=6) 24h after poly(I:C) application.

**Suppl. Fig. 7: Kinetics of macrophage and neutrophil counts in BAL fluid upon exacerbation of experimental asthma in mice**

Numbers of macrophages and neutrophils in broncho-alveolar lavage fluid (BALF) of PBS (n=8), poly(I:C) (n=8), OVA (n=8), and OVA + poly(I:C) mice (n=8) 2h, 4h, 8h, and 12h after poly(I:C) application.

**Suppl. Fig. 8: Repeated stimulation with poly(I:C) trains *IL-6* expression in airway epithelial cells *in vitro* and IL-6 release in mice with experimental asthma**

**(A)** Expression of *IL6* in BEAS-2B cells after repeated incubation with poly(I:C) after 0, 1 and 3 numbers of exposure (NOE), respectively. **(B)** IL-6 concentration relative to the respective asthmatic control group in BALF of mice repeatedly exposed to poly(I:C) for 1, 3 or 5 weeks (wk). Results are presented as mean values ± S.E.M.. Statistical significance was assessed using ordinary one-way ANOVA and Tukey´s multiple comparison post hoc analyses, **p<0.01, ***p<0.001, and ****p<0.0001.

**Suppl. Fig. 9: Methylation levels are low in IL6TS high in BEAS-2B cells**

Ward clustering of BEAS-2B cells infected with RV16 up to 5 consecutive times. Methylation levels for Illumina 450k array loci are depicted and black bars on top denote IL6TS high signature. Red box indicates samples with lower methylation.

**Suppl. Fig. 10: A low *IL6* gene methylation is associated with an increased *IL6* expression and a high IL6TS expression profile *in vitro* and in asthma patients**

**(A)** ALI-culture of primary HBEC’s. N=9 donors, stratified for each condition and for lower (<50 percentile) or higher (>50 percentile) cg015703690 methylation levels. Expression levels (arbitrary units) are the positive or negative log2 fold change of the median of the respective gene as compared to the global median. **(B)** Expression profile of interleukin 6 trans-signalling (IL6TS) genes (PDE4B, TNFAIP6, S100A9, S100A8, S100A12, CHIL3L1, IL1R2, and SPP1) in bronchial epithelial cells of patients with asthma depicted as a heat map, divided in an IL6TS high and low expression profiles, and associated with expression and methylation of the IL-6 gene (cohort I).

Results are presented as mean values ± S.E.M.. Statistical significance was assessed using two-way Anova with Sidak’s correction for multiple testing, *p<0·05.

**Suppl. Fig. 11: IL6TS high expression signature is associated with lower methylation of the *IL6* gene**

**(A)** β-values of the five published methylation loci in the *IL6* gene from Cohort I. Red boxes denote patients with lower and higher methylation. Unique patient IDs are plotted above each column of CpGs. Ward clustering (Jump 13, SAS) was performed to aggregate similar patients. Each CpG was compared in IL6TS low vs high patients. **(B)** Percentage of samples with low or high IL6TS profiles that show a low or high methylation of the *IL-6* gene, respectively (p<0·05). Results are presented as mean values ± S.E.M.. Statistical significance was assessed using Mann-Whitney, ***p<0·001. Proportions were analysed using a Chi-square-test.

**(C)-(G)**. Site-specific DNA methylation levels at selected CpG sites (B: cg1234567, C: cg00087425, D: cg05472934, E: cg07998387, F: cg15703690, G: cg01770232) in IL6TS low and IL6TS high groups. Statistical significance was assessed using Mann-Whitney test without correction for multiple testing, *p<0.1, **p<0.01.

**Suppl. Fig. 12: *IL6* gene methylation is independent of T2 endotype**

**(A)** Eosinophils percentages in Cohort I, compared between *IL6* gene methylation levels high and low. **(B)** Periostin gene expression compared between *IL6* gene methylation levels high and low. **(C)** Interleukin 1b (*IL1B)* compared between *IL6* gene methylation levels high and low. Y-axis is shown in logarithmic scale of POSTN and IL1B expression. n.s. not significant, *p<0.1, with Mann-Whitney (Jump 13, SAS, USA).

**Suppl. Fig. 13: Lower methylation of the *IL6* gene is associated with IL6TS high expression in nasal epithelial cells of children**

**(A)** Ward hierarchical clustering of Illumina 850k methylation loci of the *IL6* gene. Red box denotes lower level of methylation (β-values). Pink indicates low, light pink signifies medium and purple indicates high levels of DNA-methylation (from average β-value per CpG). **(B)** Children from the ALLIANCE cohort with matching data sets for DNA methylation and gene expression. IL6TS was sorted with Ward hierarchical clustering and high vs low compared to matching β-values of DNA-methylation. **(C)** Percentage of samples with low or high IL6TS profiles that show a low or high methylation of the IL6 gene, respectively *p<0.05

**References**

1 Heink S, Yogev N, Garbers C, et al. Trans-presentation of IL-6 by dendritic cells is required for the priming of pathogenic TH17 cells. Nat Immunol 2017; 18: 74–85. .

2 Pachynski RK, Zabel BA, Kohrt HE, *et al.* The chemoattractant chemerin suppresses melanoma by recruiting natural killer cell antitumor defenses. *Journal of Experimental Medicine* 2012; **209**: 1427–35.

3 Webering S, Lunding LP, Vock C, et al. The alpha-melanocyte-stimulating hormone acts as a local immune homeostasis factor in experimental allergic asthma. Clin Exp Allergy 2019; 49: 1026–39. .

4 Mattfeldt T, Mall G, Gharehbaghi H, Möller P. Estimation of surface area and length with the orientator. *Journal of Microscopy* 1990; **159**: 301–17.

5 Fehrenbach H, Wahlers T, Ochs M, *et al.* Ultrastructural pathology of the alveolar type II pneumocytes of human donor lungs. *Virchows Archiv* 1998; **432**: 229–39.

6 Ordoñez CL, Khashayar R, Wong HH, *et al.* Mild and Moderate Asthma Is Associated with Airway Goblet Cell Hyperplasia and Abnormalities in Mucin Gene Expression. *Am J Respir Crit Care Med* 2001; **163**: 517–23.

7 Weibel ER, Hsia CCW, Ochs M. How much is there really? Why stereology is essential in lung morphometry. *Journal of Applied Physiology* 2007; **102**: 459–67.

8 Lunding LP, Webering S, Vock C, et al. Poly(inosinic-cytidylic) acid-triggered exacerbation of experimental asthma depends on IL-17A produced by NK cells. J Immunol 2015; 194: 5615–25. .

9 Pech M, Weckmann M, König IR, *et al.* Rhinovirus infections change DNA methylation and mRNA expression in children with asthma. *PLoS One* 2018; **13**: e0205275.

10 Nemani SSP, Vermeulen CJ, Pech M, *et al.* COL4A3 expression in asthmatic epithelium depends on intronic methylation and ZNF263 binding. *ERJ Open Res* 2021; **7**: 00802–2020.

11 Vermeulen CJ, Xu C-J, Vonk JM, *et al.* Differential DNA methylation in bronchial biopsies between persistent asthma and asthma in remission. *Eur Respir J* 2020; **55**: 1901280.

Suppl. Table 1: Average sequencing library size and mapped RV16 fragments per repeated infection

| Number of Infections |  | 0 | 1 | 3 | 5 |
| --- | --- | --- | --- | --- | --- |
| Total fragments | Mean | 25530187.7 | 25405033.6 | 26328655.3 | 25267752.6 |
|  | Std Dev | 2232872.96 | 1656162.52 | 1708499.22 | 2493567.04 |
|  | N | 6 | 5 | 4 | 5 |
| Mapped fragments (RV16) | Mean | 0 | 2.1 | 1.25 | 3.6 |
|  | Std Dev | 0.0 | 1.6 | 1.9 | 5.0 |
| min – max fragments | min-max | 0 | 0-6 | 0-4 | 0-9 |
|  | N | 6 | 5 | 4 | 5 |

Suppl. Table 2: Average sequencing library size and mapped RV16 fragments per Mock control for each repeated infection step.

| Number of Infections (Mock) |  | 0 | 1 | 3 | 5 |
| --- | --- | --- | --- | --- | --- |
| Total fragments | Mean | 24875070.5 | 27155865.5 | 25980154 | 23139100 |
|  | N | 2 | 2 | 1 | 1 |
| Mapped fragments (RV16) | Mean | 0 | 0 | 0 | 0 |
|  | N | 2 | 2 | 1 | 1 |
